# Gene-Expression Profiling of Mucinous Ovarian Tumors and Comparison with Upper and Lower Gastrointestinal Tumors Identifies Markers Associated with Adverse Outcomes

**DOI:** 10.1158/1078-0432.CCR-22-1206

**Published:** 2022-10-07

**Authors:** Nicola S. Meagher, Kylie L. Gorringe, Matthew Wakefield, Adelyn Bolithon, Chi Nam Ignatius Pang, Derek S. Chiu, Michael S. Anglesio, Kylie-Ann Mallitt, Jennifer A. Doherty, Holly R. Harris, Joellen M. Schildkraut, Andrew Berchuck, Kara L. Cushing-Haugen, Ksenia Chezar, Angela Chou, Adeline Tan, Jennifer Alsop, Ellen Barlow, Matthias W. Beckmann, Jessica Boros, David D.L. Bowtell, for the AOCS Group, Alison H. Brand, James D. Brenton, Ian Campbell, Dane Cheasley, Joshua Cohen, Cezary Cybulski, Esther Elishaev, Ramona Erber, Rhonda Farrell, Anna Fischer, Zhuxuan Fu, Blake Gilks, Anthony J. Gill, for the Australian Pancreatic Genome Initiative, Charlie Gourley, Marcel Grube, Paul R. Harnett, Arndt Hartmann, Anusha Hettiaratchi, Claus K. Høgdall, Tomasz Huzarski, Anna Jakubowska, Mercedes Jimenez-Linan, Catherine J. Kennedy, Byoung-Gie Kim, Jae-Weon Kim, Jae-Hoon Kim, Kayla Klett, Jennifer M. Koziak, Tiffany Lai, Angela Laslavic, Jenny Lester, Yee Leung, Na Li, Winston Liauw, Belle W.X. Lim, Anna Linder, Jan Lubiński, Sakshi Mahale, Constantina Mateoiu, Simone McInerny, Janusz Menkiszak, Parham Minoo, Suzana Mittelstadt, David Morris, Sandra Orsulic, Sang-Yoon Park, Celeste Leigh Pearce, John V. Pearson, Malcolm C. Pike, Carmel M. Quinn, Ganendra Raj Mohan, Jianyu Rao, Marjorie J. Riggan, Matthias Ruebner, Stuart Salfinger, Clare L. Scott, Mitul Shah, Helen Steed, Colin J.R. Stewart, Deepak Subramanian, Soseul Sung, Katrina Tang, Paul Timpson, Robyn L. Ward, Rebekka Wiedenhoefer, Heather Thorne, for the kConFab Investigators, Paul A. Cohen, Philip Crowe, Peter A. Fasching, Jacek Gronwald, Nicholas J. Hawkins, Estrid Høgdall, David G. Huntsman, Paul A. James, Beth Y. Karlan, Linda E. Kelemen, Stefan Kommoss, Gottfried E. Konecny, Francesmary Modugno, Sue K. Park, Annette Staebler, Karin Sundfeldt, Anna H. Wu, Aline Talhouk, Paul D.P. Pharoah, Lyndal Anderson, Anna DeFazio, Martin Köbel, Michael L. Friedlander, Susan J. Ramus

**Affiliations:** 1 School of Clinical Medicine, Faculty of Medicine and Health, University of NSW Sydney, Sydney, New South Wales, Australia.; 2 Adult Cancer Program, Lowy Cancer Research Centre, University of NSW Sydney, Sydney, New South Wales, Australia.; 3 Peter MacCallum Cancer Centre, Melbourne, Victoria, Australia.; 4 Sir Peter MacCallum Department of Medical Oncology, The University of Melbourne, Parkville, Victoria, Australia.; 5 The Walter and Eliza Hall Institute of Medical Research, Parkville, Victoria, Australia.; 6 Department of Medical Biology, The University of Melbourne, Melbourne, Victoria, Australia.; 7 Department of Obstetrics and Gynaecology, The University of Melbourne, Melbourne, Victoria, Australia.; 8 School of Biotechnology and Biomolecular Sciences, The University of New South Wales, Sydney, New South Wales, Australia.; 9 Bioinformatics Unit, Children's Medical Research Institute, Westmead, Sydney, Australia.; 10 British Columbia's Gynecological Cancer Research Team (OVCARE), University of British Columbia, BC Cancer, and Vancouver General Hospital, Vancouver, British Columbia, Canada.; 11 Department of Obstetrics and Gynecology, University of British Columbia, Vancouver, British Columbia, Canada.; 12 Centre for Big Data Research in Health, University of New South Wales Sydney, Sydney, New South Wales, Australia.; 13 Huntsman Cancer Institute, Department of Population Health Sciences, University of Utah, Salt Lake City, Utah.; 14 Program in Epidemiology, Division of Public Health Sciences, Fred Hutchinson Cancer Center, Seattle, Washington.; 15 Department of Epidemiology, University of Washington, Seattle, Washington.; 16 Department of Epidemiology, Rollins School of Public Health, Emory University, Atlanta, Georgia.; 17 Department of Obstetrics and Gynecology, Division of Gynecologic Oncology, Duke University Medical Center, Durham, North Carolina.; 18 Department of Pathology and Laboratory Medicine, University of Calgary, Foothills Medical Center, Calgary, Alberta, Canada.; 19 The Kinghorn Cancer Centre, Garvan Institute of Medical Research, Sydney, New South Wales, Australia.; 20 Department of Anatomical Pathology, Royal North Shore Hospital, Sydney, New South Wales, Australia.; 21 The University of Sydney, Sydney, New South Wales, Australia.; 22 Division of Obstetrics and Gynaecology, Medical School, University of Western Australia, Crawley, Western Australia, Australia.; 23 Western Women's Pathology, Western Diagnostic Pathology, Wembley, Western Australia, Australia.; 24 Centre for Cancer Genetic Epidemiology, Department of Oncology, University of Cambridge, Cambridge, United Kingdom.; 25 Gynaecological Cancer Centre, Royal Hospital for Women, Sydney, New South Wales, Australia.; 26 Department of Gynecology and Obstetrics, Comprehensive Cancer Center Erlangen-EMN, Friedrich-Alexander University Erlangen-Nuremberg, University Hospital Erlangen, Erlangen, Germany.; 27 Centre for Cancer Research, The Westmead Institute for Medical Research, Sydney, New South Wales, Australia.; 28 Department of Gynaecological Oncology, Westmead Hospital, Sydney, New South Wales, Australia.; 29 Cancer Research UK Cambridge Institute, University of Cambridge, Cambridge, United Kingdom.; 30 David Geffen School of Medicine, Department of Obstetrics and Gynecology, University of California at Los Angeles, Los Angeles, California.; 31 Department of Genetics and Pathology, International Hereditary Cancer Center, Pomeranian Medical University, Szczecin, Poland.; 32 Department of Pathology, University of Pittsburgh School of Medicine, Pittsburgh, Pennsylvania.; 33 Institute of Pathology, Comprehensive Cancer Center Erlangen-EMN, Friedrich-Alexander Universität Erlangen-Nürnberg, University Hospital Erlangen, Erlangen, Germany.; 34 Prince of Wales Private Hospital, Randwick, New South Wales, Australia.; 35 Institute of Pathology and Neuropathology, Tübingen University Hospital, Tübingen, Germany.; 36 Department of Epidemiology, University of Pittsburgh School of Public Health, Pittsburgh, Pennsylvania.; 37 Department of Pathology and Laboratory Medicine, University of British Columbia, Vancouver, British Columbia, Canada.; 38 Nicola Murray Centre for Ovarian Cancer Research, Cancer Research UK Scotland Centre, University of Edinburgh, Edinburgh, United Kingdom.; 39 Department of Women's Health, Tübingen University Hospital, Tübingen, Germany.; 40 Crown Princess Mary Cancer Centre, Westmead Hospital, Sydney, New South Wales, Australia.; 41 The Health Precincts Biobank (formerly the Health Science Alliance Biobank), UNSW Biospecimen Services, Mark Wainwright Analytical Centre, University of New South Wales Sydney, Sydney, New South Wales, Australia.; 42 Department of Gynaecology, Rigshospitalet, University of Copenhagen, Copenhagen, Denmark.; 43 Department of Genetics and Pathology, University of Zielona Góra, Zielona Góra, Poland.; 44 Independent Laboratory of Molecular Biology and Genetic Diagnostics, Pomeranian Medical University, Szczecin, Poland.; 45 Department of Histopathology, Addenbrooke's Hospital, Cambridge, United Kingdom.; 46 Department of Obstetrics and Gynecology, Samsung Medical Center, Sungkyunkwan University School of Medicine, Seoul, Korea.; 47 Department of Obstetrics and Gynecology, Seoul National University College of Medicine, Seoul, Korea.; 48 Department of Obstetrics and Gynecology, Gangnam Severance Hospital, Yonsei University College of Medicine, Seoul, Republic of Korea.; 49 Women's Cancer Research Center, Magee-Womens Research Institute and Hillman Cancer Center, Pittsburgh, Pennsylvania.; 50 Alberta Health Services-Cancer Care, Calgary, Alberta, Canada.; 51 Division of Gynecologic Oncology, Department of Obstetrics, Gynecology and Reproductive Sciences, University of Pittsburgh School of Medicine, Pittsburgh, Pennsylvania.; 52 Department of Gynaecological Oncology, King Edward Memorial Hospital, Subiaco, Western Australia, Australia.; 53 Australia New Zealand Gynaecological Oncology Group, Camperdown, New South Wales, Australia.; 54 Parkville Familial Cancer Centre, The Royal Melbourne Hospital and Peter MacCallum Cancer Centre, Melbourne, Victoria, Australia.; 55 Cancer Care Centre, St George Hospital, Sydney, New South Wales, Australia.; 56 Department of Obstetrics and Gynecology, Inst of Clinical Science, Sahlgrenska Center for Cancer Research, University of Gothenburg, Gothenburg, Sweden.; 57 Department of Pathology, Sahlgrenska University Hospital, Gothenburg, Sweden.; 58 Department of Gynecological Surgery and Gynecological Oncology of Adults and Adolescents, Pomeranian Medical University, Szczecin, Poland.; 59 St George and Sutherland Clinical School, University of New South Wales Sydney, Sydney, New South Wales, Australia.; 60 Center for Gynecologic Cancer, National Cancer Center Institute for Cancer Control, Goyang, Republic of Korea.; 61 Department of Epidemiology, University of Michigan School of Public Health, Ann Arbor, Michigan.; 62 Department of Preventive Medicine, Keck School of Medicine, University of Southern California Norris Comprehensive Cancer Center, Los Angeles, California.; 63 QIMR Berghofer Medical Research Institute, Brisbane, Queensland, Australia.; 64 Department of Epidemiology and Biostatistics, Memorial Sloan-Kettering Cancer Center, New York, New York.; 65 Department of Gynaecological Oncology, St John of God Subiaco Hospital, Subiaco, Western Australia, Australia.; 66 School of Medicine, University of Notre Dame, Fremantle, Western Australia, Australia.; 67 Department of Pathology and Laboratory Medicine, David Geffen School of Medicine, University of California Los Angeles, Los Angeles, California.; 68 Division of Gynecologic Oncology, Department of Obstetrics and Gynecology, University of Alberta, Edmonton, Alberta, Canada.; 69 Section of Gynecologic Oncology Surgery, North Zone, Alberta Health Services, Edmonton, Alberta, Canada.; 70 School for Women's and Infants' Health, University of Western Australia, Perth, Western Australia, Australia.; 71 Department of Biomedical Sciences, Seoul National University Graduate School, Seoul, Korea.; 72 Cancer Research Institute, Seoul National University, Seoul, Korea.; 73 Department of Preventive Medicine, Seoul National University College of Medicine, Seoul, Korea.; 74 Department of Anatomical Pathology, Prince of Wales Hospital, Sydney, New South Wales, Australia.; 75 Department of Gynaecological Oncology, St John of God Subiaco Hospital, Subiaco, Western Australia, Australia.; 76 Department of Surgery, Prince of Wales Private Hospital, Randwick, New South Wales, Australia.; 77 Department of Pathology, Herlev Hospital, University of Copenhagen, Copenhagen, Denmark.; 78 Department of Molecular Oncology, BC Cancer Research Centre, Vancouver, British Columbia, Canada.; 79 Hollings Cancer Center, Medical University of South Carolina, Charleston, South Carolina.; 80 Integrated Major in Innovative Medical Science, Seoul National University College of Medicine, Seoul, South Korea.; 81 Centre for Cancer Genetic Epidemiology, Department of Public Health and Primary Care, University of Cambridge, Cambridge, United Kingdom.; 82 Department of Tissue Pathology and Diagnostic Oncology, Royal Prince Alfred Hospital and NSW Health Pathology, Sydney, New South Wales, Australia.; 83 The Daffodil Centre, a joint venture with Cancer Council NSW, The University of Sydney, Sydney, New South Wales, Australia.; 84 Nelune Comprehensive Cancer Centre, Prince of Wales Hospital, Sydney, New South Wales, Australia.

## Abstract

**Purpose:**

Advanced-stage mucinous ovarian carcinoma (MOC) has poor chemotherapy response and prognosis and lacks biomarkers to aid stage I adjuvant treatment. Differentiating primary MOC from gastrointestinal (GI) metastases to the ovary is also challenging due to phenotypic similarities. Clinicopathologic and gene-expression data were analyzed to identify prognostic and diagnostic features.

**Experimental Design:**

Discovery analyses selected 19 genes with prognostic/diagnostic potential. Validation was performed through the Ovarian Tumor Tissue Analysis consortium and GI cancer biobanks comprising 604 patients with MOC (*n* = 333), mucinous borderline ovarian tumors (MBOT, *n* = 151), and upper GI (*n* = 65) and lower GI tumors (*n* = 55).

**Results:**

Infiltrative pattern of invasion was associated with decreased overall survival (OS) within 2 years from diagnosis, compared with expansile pattern in stage I MOC [hazard ratio (HR), 2.77; 95% confidence interval (CI), 1.04–7.41, *P* = 0.042]. Increased expression of *THBS2* and *TAGLN* was associated with shorter OS in MOC patients (HR, 1.25; 95% CI, 1.04–1.51, *P* = 0.016) and (HR, 1.21; 95% CI, 1.01–1.45, *P* = 0.043), respectively. *ERBB2* (HER2) amplification or high mRNA expression was evident in 64 of 243 (26%) of MOCs, but only 8 of 243 (3%) were also infiltrative (4/39, 10%) or stage III/IV (4/31, 13%).

**Conclusions:**

An infiltrative growth pattern infers poor prognosis within 2 years from diagnosis and may help select stage I patients for adjuvant therapy. High expression of *THBS2* and *TAGLN* in MOC confers an adverse prognosis and is upregulated in the infiltrative subtype, which warrants further investigation. Anti-HER2 therapy should be investigated in a subset of patients. MOC samples clustered with upper GI, yet markers to differentiate these entities remain elusive, suggesting similar underlying biology and shared treatment strategies.

**Translational Relevance**

Mucinous ovarian cancer (MOC) is a rare histologic subtype of epithelial ovarian cancer, lacking prognostic markers in stage I tumors, with poor prognosis and low response to chemotherapy at advanced stage. Phenotypic similarities between MOC and lower and upper gastrointestinal (GI) tumors create diagnostic challenges when they spread to the ovary. In the largest series to date of stage I MOC characterized pathologically by a pattern of invasion, we confirm that an infiltrative pattern is a poor prognostic factor, supporting consideration of adjuvant chemotherapy. We identified two prognostic markers, *THBS2* and *TAGLN*, in MOC worthy of further investigation. Despite a higher frequency of HER2^+^ in low-stage and expansile pattern MOC, just 3% of patients with HER2^+^ MOC have a poor prognosis (advanced stage or infiltrative) and should be considered for anti-HER2 therapy. Comparisons with GI cancers at the mRNA expression level conclude that the distinction between pancreatic and gastric cancers remains a challenge.

## Introduction

Mucinous ovarian carcinoma (MOC) is a rare histologic type that is less well characterized compared with more common ovarian cancer histotypes. A clinical problem frequently encountered in patients diagnosed with advanced-stage MOC is the uncertainty as to whether the primary cancer is ovarian or metastatic from other sites. Metastases typically originate from the gastrointestinal (GI) tract, and the primary tumor may not be evident at surgery or on imaging (1–3). Earlier literature has focused on differentiating MOC from lower GI tumors, due to the relatively high frequency of reclassification from “primary MOC” to primary colorectal or appendiceal neoplasms metastatic to the ovary following expert pathologic review (1). Gene and protein expression studies have led to improved diagnostic algorithms for lower GI tumors (4), but robust markers to differentiate primary MOC from metastases of upper GI origin are lacking (5).

Patients with MOC diagnosed at an advanced stage (International Federation of Gynecology and Obstetrics (FIGO), stage III/IV) have very poor survival (5-year survival 15%) (6). Treatment guidelines for FIGO Stage IC–IV MOC are primary cytoreductive surgery and adjuvant chemotherapy with carboplatin and paclitaxel (± bevacizumab), similar to the treatment of patients with more common ovarian cancer histotypes (7). However, given the poor outcomes of patients with advanced-stage MOC, there is a great need for more effective treatment strategies. This has proven to be difficult due to the rarity of MOC, and difficulties in making a definitive diagnosis based on routine histopathology. The only randomized trial designed to compare carboplatin and paclitaxel (± bevacizumab) with a GI chemotherapy regimen, capecitabine and oxaliplatin (± bevacizumab) for MOC was closed prematurely (8). The major obstacles were a limited number of sites participating due to the cost of opening trials with low accrual, and the high frequency of misclassified GI metastases on central pathology review (8). The United States National Comprehensive Cancer Network (US NCCN) guidelines now recommend either ovarian or GI regimens for patients with MOC based on expert opinion and small retrospective series but the evidence base is low (9–11). A better understanding of the molecular differences and similarities between MOC and mucinous carcinomas arising in the GI tract is needed. This could guide treatment recommendations and inform the design of future basket clinical trials that include advanced-stage mucinous cancers irrespective of the site of origin.

For most patients diagnosed with stage I MOC (∼70%–80% of all MOC), prognosis is good; however, the clinical challenge is identifying the subset of patients with a higher mortality risk. Notwithstanding the limited evidence for efficacy, the US NCCN guidelines recommend adjuvant chemotherapy for MOC FIGO stage IC or higher(9), whereas the European guidelines include consideration of adjuvant chemotherapy for patients with FIGO stage IA or IB MOC with an infiltrative growth pattern (12). This pathologic feature exhibits destructive invasion of haphazardly arranged and angulated tumor cell nests into a desmoplastic stroma (13) and has been suggested to confer an increased risk of relapse and mortality. This contrasts with expansile invasion characterized by complex tumor nodules with confluent epithelial growth (14). Published series to date in stage I MOC have reported inconsistent results and are limited by small sample sizes (*n* = 21–64; refs. 15–20). Determining the role of a pattern of invasion in a large stage I MOC cohort is needed to help inform treatment recommendations if a higher risk of recurrence is confirmed.

We analyzed clinical, pathologic and gene-expression data, in tumor samples from a large cohort of patients with MOC. We aimed to identify new prognostic biomarkers, as well as validate the prognostic association between the pattern of invasion and survival in a well-powered, adjusted analysis. We also aimed to differentiate MOC from primary and metastatic GI cancers based on mRNA expression of key genes, or to identify shared markers that may help select targeted therapeutic options independent of the site of origin.

## Materials and Methods

### Patient cohort

Samples and data were submitted from 848 patients diagnosed with ovarian or GI tumors. These were from 24 sites from the Ovarian Tumor Tissue Analysis consortium, the Australian Pancreatic Genome Initiative, the Molecular and Cellular Oncology colorectal biobank (UNSW) and the Department of Pathology, University of Calgary. Clinical data including patient age at diagnosis, tumor stage, histopathologic grade, and overall survival (OS) were provided by the respective studies. The study was approved by the UNSW Human Research Ethics Committee (approval HC17182), all contributing sites obtained written informed patient consent or had relevant ethical/institutional review board approval for waiver of consent, and all studies were conducted in accordance with recognized ethical guidelines (Supplementary Table S1).

Hematoxylin and eosin (H&E)-stained slides were reviewed to confirm the diagnosis, identify the anatomic site of the tissue sample used in this study, mark the region for RNA extraction, estimate the percentage of tumor cells within the extraction area, and classify the pattern of invasion. A centralized pathology review was performed by expert gynecologic or GI pathologists (MK, LA, AT, NH, and AC). An infiltrative pattern of invasion in MOC was classified with a linear extent of stromal invasion >5 mm (21). Samples from 178 patients were excluded (Supplementary Fig. S1) due to low (<20%) tumor cellularity (*n* = 52), ineligible diagnosis following pathology review, including “seromucinous” tumors (*n* = 55), unknown or unclassifiable discordant diagnosis (*n* = 54; Supplementary Table S2), or no tumor in the block (*n* = 17). For 77 cases with 2 or more slides suitable for inclusion, the slide with the most representative and/or highest tumor cellularity was selected. Following RNA extraction, another 36 samples with a yield less than 32 ng/μL were excluded.

RNA samples from a total of 634 patients were eligible for the NanoString Plexset assay, extracted from either formalin-fixed, paraffin-embedded (FFPE) whole sections (*n* = 403), FFPE cores (*n* = 191), or fresh-frozen sections (*n* = 40). Samples from the prognostic gene discovery analysis were excluded from validation analyses to preclude overfitting of the data (*n* = 54). A second sample was analyzed in a subset of 33 patients: either multiple blocks from the same tumor or multiple tumor tissue sites.

### Gene selection

We analyzed two data sets to select 19 genes of potential prognostic or diagnostic value in MOC. Candidate prognostic genes were identified based on analysis of 513 genes run on a NanoString platform (Supplementary Appendix S1; Supplementary Methods). The data set included 60 MOCs among a study of predominantly high-grade serous ovarian cancers that have been published elsewhere (22, 23). We identified four genes (*THBS2, TAGLN, DCN,* and *PLA2R1*) that were differentially expressed between low (I/II, *n* = 49) and high (III/IV, *n* = 11) stage MOC (Supplementary Methods Table SA), and increased expression of three of these (*THBS2, TAGLN, DCN*) were associated with a poorer OS on univariate analysis (Supplementary Methods, Table SB).

Candidate diagnostic classification genes [*MUC16* (encoding CA125), *GKN1*, *PGC*, *MEP1A*, *KRT20* (encoding CK20), *MUC5AC*, *CLDN18*, *VSIG1*, and *ANXA10*] were from an analysis by the Genomic Analysis of Mucinous Tumours (GAMuT) study (24), whereby an exploratory RNA-seq cluster analysis was performed to differentiate between benign mucinous ovarian tumors, mucinous borderline ovarian tumors (MBOT), MOC, and upper and lower GI metastases to the ovary (Supplementary Methods). The goal was to identify differential markers between entities with biological plausibility and available antibodies for future potential validation by IHC. We selected six additional genes (*ERBB2, TYMS, SATB2, MUC2, PD-1*, and *PD-L1*) for diagnostic or therapeutic interest from the literature (4, 25–27). Housekeeping genes (*DNAH6*, *LDHA*, *MTG1*, *POLR1B*, and *TBP*) were selected based on consistent expression across different cancer types using publicly available TCGA RNA-seq data for colorectal adenocarcinomas (COAD), ovarian (OV), pancreas (PAAD), stomach (STAD), and in the GAMuT RNA-seq data set for mucinous histology (Supplementary Methods).

### NanoString PlexSet assay

Extraction of RNA and sample preparation for the NanoString assay was as described previously (22, 23). A Plexset-24 assay of 24 customized probes (Supplementary Table S3) was used and due to the multiplex design, one patient sample with adequate quantity was selected as an internal calibrator. The assay was run by the Ramaciotti Centre for Genomics (UNSW Sydney, Australia).

### Data quality assurance and normalization

We performed single-sample data normalization as previously described (28), with adjustments to account for the Plexset assay. Raw counts were normalized to the housekeeping genes and then to the calibrator sample. Expression of the housekeeping gene *DNAH6* was at the limit of detection, and the data were therefore excluded. We transformed the normalized gene-expression data by taking the logarithm with base 2. Quality control (QC) measures were assessed by sample, by codeset and by cartridge to examine relevant levels of variability. Measures included the signal-to-noise ratio (SNR <150), percentage of genes detected (above background plus two standard deviations), and expected expression of housekeeping genes.

### IHC and silver *in situ* hybridization (SISH)

We performed ERBB2/HER2 IHC using anti-HER2/neu (4B5), Roche Diagnostics (6 μg/mL) and SISH using HER2/Ch17 Dual ISH DNA Probe Cocktail, Roche Diagnostics, concentration (14.24 μg/mL). Staining was performed on the Ventana Benchmark ULTRA Platform on 4-μm tissue microarray sections for a subset of cases from one study (WMH). For ERBB2/HER2 IHC, we used serous endometrial scoring guidelines (29) and a score of 3+ was given where >30% tumor cells showed intense complete membrane or basolateral membrane staining. Positive amplification was defined as either clusters (signal in >20 cells) or HER2/CEP17 ratio ≥2 or >6 copies/nucleus and IHC 2+.

### Statistical analysis

OS was estimated using Cox proportional hazards, with right censoring at 10 years, and left truncation of prevalent cases. Validation of the association between gene expression and survival for the 4 candidate prognostic genes (*THBS2*, *TAGLN*, *DCN*, and *PLA2R1*) was limited to new cases, removing the 54 overlapping samples from the discovery data set. All multivariable analyses were adjusted for age and tumor stage and stratified by study site. Survival analyses of gene-expression data used continuous normalized mRNA expression, examining one gene per model. The proportional hazards assumption was tested using the cox.zph function in the survival package in R. Survival curves were produced using the Kaplan–Meier method. For visualization, survival curves of expression by tertile for significant genes were plotted. A time-dependent analysis was performed to assess the pattern of invasion in MOC (all stages and stage I alone) using the survSplit function in R (30), with stratification applied at 0 to 2 years versus >2 years based on an inspection of the survival curves. This was run with and without left truncation to ensure consistent results for the time from diagnosis as well as from the study entry. Comparisons of gene expression between groups were performed using either the Student *t* test for 2 group comparisons or one-way ANOVA with Tukey post hoc test for multiple comparisons. Correlation between mRNA expression and IHC scores for ERBB2/HER2 were calculated with Spearman correlation coefficients. Correlation between the expression of all 19 candidate genes in different tumor blocks from the same patient was calculated using the Pearson correlation coefficient. All statistical analyses were performed using R v4.1.2.

We performed all analyses of gene-expression data on samples where the original diagnosis was concordant with the pathology review of the tissue being run on the assay to avoid misclassification.

### Bioinformatics analysis

We used unsupervised hierarchical clustering and clustered samples based on gene-expression profiles. We used the “complete” agglomeration method and measured the Euclidean distance between samples. The heat maps were drawn using the iheatmapr package (v0.5.1) in R (31). Diagnosis groups in the clustering were MBOT, low stage (I/II) MOC, advanced stage (III/IV) MOC, pancreas, gastric, and lower GI (colorectal and appendiceal combined). We used random forest analysis and stratified bootstrapping (32) to assess the ability of the gene-expression profiles to predict the disease class (diagnosis group) of each sample. The cohort was divided into independent training and testing sets using stratified random subsampling, maintaining a balanced proportion of samples of each disease class. The training data set was used to train a random forest classifier (the randomForest package in R, version 4.6-14) using default parameters and the classifier was benchmarked against the test set to obtain an error rate (Supplementary Methods). We repeated the above analyses 100 times to obtain a distribution of error rates, the mean overall error rate, and the mean and standard deviation of each element of the confusion matrix, to tabulate the number of samples associated with the actual and predicted class.

### Data availability

The data generated in this study are publicly available in the Gene-Expression Omnibus (GEO; accession number GSE203611).

## Results

### Patient cohort

We generated RNA expression for 19 candidate genes from 634 patients, on a NanoString Plexset assay, of which one patient sample was used as a calibrator and excluded from further analysis. Technical replicates (*n* = 13) showed high correlation (intraclass correlation coefficient range, 0.94–0.99). Following data processing, 29 samples failed QC and were excluded. Fifty-four samples and seven genes overlapped the discovery NanoString data set and the Plexset, and the observed adjusted intraclass coefficient was 0.69 (median R = 0.90, range, 0.34 *PD-L1* to 0.98 *ERBB2*). The final analytic cohort of 604 patients was divided into four diagnostic groups, MOC (*n* = 333), MBOT (*n* = 151), upper GI (*n* = 65), and lower GI (*n* = 55; Table 1). Of the 333 MOCs, 226 were low stage (I/II; 86% of cases with known stage). Upper GI included primary and metastatic pancreatic ductal adenocarcinoma (PDAC), intraductal papillary mucinous neoplasms (IPMN) with invasion, pancreatic mucinous cystadenocarcinomas, and gastric adenocarcinomas. Lower GI included primary and metastatic mucinous and nonmucinous colorectal and appendiceal tumors.

**Table 1.** Patient characteristics and analytical cohorts.

	MOC		MBOT		Upper GI^a^		Lower GI^b^	
Clinical and gene-expression data *n* = 604	333		151		65		55	
**Age at diagnosis (years)**								
Median	53		47		67		68	
Range	18–95		18–91		31–85		39–89	
	***n***	**% of known**	***n***	**% of known**	***n***	**% of known**	***n***	**% of known**
**Stage**								
I	206	78%	98	95%	5	8%	8	18%
II	20	8%	2	2%	26	41%	11	25%
III	31	12%	3	3%	26	41%	15	34%
IV	7	3%	0	0%	6	10%	10	23%
Unknown	69		48		2		11	
**Sex**								
Female	333	100%	151	100%	33	51%	47	85%
Male	0	0%	0	0%	32	49%	8	15%
**Grade**								
1	136	46%	n/a		3	5%	12	23%
2	113	38%	n/a		44	72%	32	62%
3	46	16%	n/a		14	23%	8	15%
Unknown	38		n/a		4		3	
**Residual disease**								
Nil macroscopic	145	86%	62	90%	18	82%	25	84%
Yes	24	14%	7	10%	4	18%	7	25%
Unknown	164		82		43		23	

Abbreviations: MOC, mucinous ovarian carcinoma; MBOT, mucinous borderline ovarian tumor; GI, gastrointestinal.

^a^Pancreas cancer (*n* = 57); gastric cancer (*n* = 5); upper GI metastases, unknown primary (*n* = 3).

^b^Colorectal cancer (*n* = 36); appendiceal cancer (*n* = 15); lower GI metastases, unknown primary (*n* = 4).

### Pathology review concordance and data analysis

**Figure 1.**
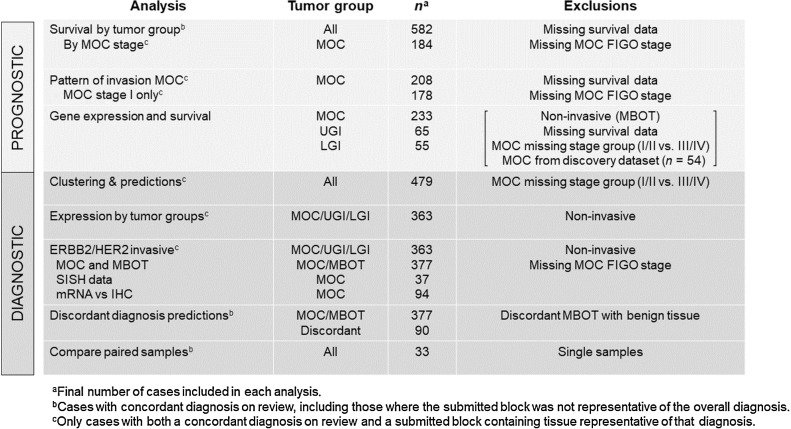
Schema of study numbers for each analysis to describe different cohort numbers due to pathology review and missing data. MOC, mucinous ovarian carcinoma; MBOT, mucinous borderline ovarian tumor; LGI, lower gastrointestinal; UGI, upper gastrointestinal; SISH, silver *in situ* hybridization.

Pathology review found that 107 of the 604 cases were discordant between the original diagnosis and the review diagnosis of the sample run on the Plexset (Supplementary Table S4). Given the known intratumoral pathologic heterogeneity of large mucinous ovarian tumors, and the focal nature of some MOC, we considered that these may be cases where the tissue submitted was not representative of the overall patient diagnosis (e.g., a block from a MOC case that contains only mucinous borderline tumor tissue). These patients were included in survival analysis that were unrelated to specific tissue features, based on their highest pathologic diagnosis. For analyses involving features of the tissue itself (pattern of invasion and gene expression), we only included the concordant cases (*n* = 497) to avoid misclassification. For each analysis, samples with missing clinical data were also removed where relevant, while attempting to maximize the sample size in this rare histotype (Fig. 1).

### Prognosis

#### OS by tumor group

**Figure 2.**
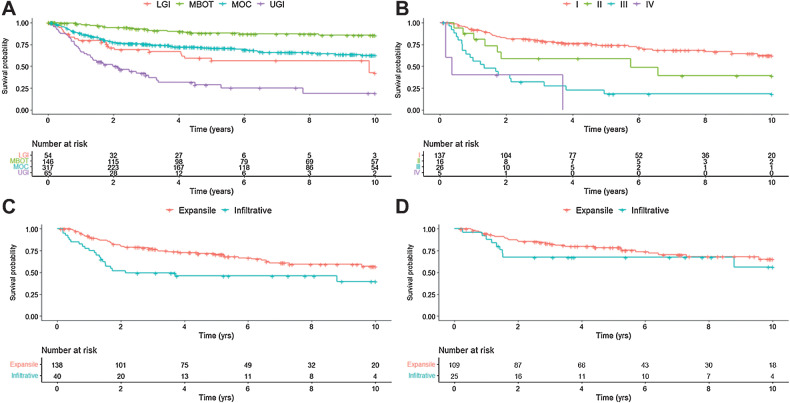
Kaplan–Meier curves of OS in (**A**) main tumor groups (*n* = 582)—MBOT, MOC, LGI, and UGI; (**B**) patients with MOC by FIGO stage (*n* = 184); (**C**) patients with MOC by pattern of invasion in all stages (*n* = 178); and (**D**) patients with stage I MOC (*n* = 134), by pattern of invasion.

Survival analysis included all patients with a concordant diagnosis and those where a nonrepresentative block was submitted (604 patients), of which 582 had complete clinical data. The 5-year unadjusted OS was highest in MBOT (88%), intermediate for MOC (71%), and considerably lower for lower GI (56%) and upper GI (29%, log-rank *P* < 0.0001; Fig. 2A). We also examined OS in MOC by FIGO stage (*n* = 184) and observed decreasing OS with increasing FIGO stage (*P* < 0.0001; Fig. 2B)

#### Pattern of invasion in mucinous ovarian carcinoma

The pattern of invasion was available for 208 MOC cases, with 167 (80%) classified as expansile and 41 (20%) as infiltrative. The proportion of cases with an infiltrative pattern increased with more advanced stage, 18% of stage I MOC had an infiltrative pattern, as did 27% of stage II, 29% of stage III, and 80% of stage IV MOC (Supplementary Fig. S2). Of the cases with FIGO stage data, 178 had survival data. Univariate survival analysis demonstrated that an infiltrative growth pattern was associated with poorer OS (HR, 2.20; 1.33–3.64; *P* < 0.01; Table 2 and Fig. 2C); however, multivariable modeling adjusting for age, stage, and stratified by study site violated the proportional hazards assumption, suggestive of a time-dependent association. A time-split analysis was performed for the periods 0–2 years and >2 years after diagnosis based on inspection of the survival curves. This showed a significant time-dependent association between infiltrative growth pattern and poorer OS at 0–2 years after diagnosis (adj-HR 3.06; 1.49–6.29; *P* = 0.002), but was not significant during the period >2 years (*P* = 0.297). Similarly, within stage I MOC (*n* = 134), the Kaplan–Meier curves showed that most deaths in the infiltrative type occurred within the first 2 years of diagnosis (Fig. 2D). A significant association between infiltrative subtype and poorer OS in stage I MOC was observed within the first 2 years following diagnosis (adj-HR 2.77; 1.04–7.41; *P* = 0.042; Table 2).

**Table 2.** Overall survival in MOC by pattern of invasion.

				Univariate		Multivariable^a^	
Analyses		*n*	Deaths	HR (95% CI)	*P*	HR (95% CI)	*P*
**All stages**	Expansile	138	45	Ref		Ref	
	**Infiltrative**	**40**	**23**	**2.20 (1.33–3.64)**	**0.002**	**1.86 (1.02–3.42)**	**0.044**
Stratification by time							
0–2 years	Expansile	138	25	Ref		Ref	
	**Infiltrative**	**40**	**19**	**3.12 (1.72–5.67)**	**1.88E-04**	**3.06 (1.49–6.29)**	**0.002**
>2 years	Expansile	101	20	Ref		Ref	
	Infiltrative	20	4	0.71 (0.21–2.39)	0.580	0.45 (0.10–2.03)	0.297
**Stage I**	Expansile	109	28	Ref		Ref	
	Infiltrative	25	10	1.52 (0.71–3.21)	0.278	1.40 (0.59–3.33)	0.447
Stratification by time							
0–2 years	Expansile	108	14	Ref		Ref	
	**Infiltrative**	24	**8**	**2.67 (1.12–6.37)**	**0.027**	**2.77 (1.04–7.41)**	**0.042**
>2 years	Expansile	87	14	Ref		Ref	
	Infiltrative	16	2	0.34 (0.04–2.60)	0.299	0.34 (0.04–2.69)	0.309

^a^All stage multivariable analysis adjusted for age and stage, stratified by site; stage I multivariable analysis adjusted for age and site.

Of the 19 genes analyzed, 12 had a statistically significant difference in mean expression between expansile and infiltrative subtypes (*n* = 208; Supplementary Fig. S3). Eight genes were significantly higher in infiltrative (*THBS2*, *TAGLN*, *DCN*, *SATB2*, *GKN1*, *MUC16*, *PLA2R1*, and *MUC2*), and the expansile subtype had significantly higher expression of *ERBB2*, *PGC, ANXA10*, and *CLDN18* (Supplementary Fig. S3). In FIGO stage I cases (*n* = 134), 6 of these genes were higher in the infiltrative subtype (*THBS2*, *TAGLN*, *DCN*, *PLA2R1, SATB2*, and *GKN1*), and 1 higher in expansile (*ERBB2*; Supplementary Fig. S3).

#### Gene expression and OS

We assessed the association between gene expression and survival in 233 MOC patients. Univariate analysis found five genes associated with OS—*THBS2*, *TAGLN*, *DCN*, *PLA2R1, ERBB2* (Table 3). After adjusting for age and stage and stratifying by study site, increased expression of two genes was associated with poorer OS: *THBS2*, HR 1.25 (95% CI, 1.04–1.51), *P* = 0.016 and *TAGLN* 1.21 (1.01–1.45), *P* = 0.043. We plotted tertiles of expression for each gene for visualization (Supplementary Fig. S4). These two genes were also upregulated in tumors with an infiltrative pattern of invasion (Supplementary Fig. S5).

**Table 3.** Associations between gene expression and stage group and OS in MOC.

	Mean expression by stage group				Univariate			Multivariable	
Gene	I/II (*n* = 189)	III/IV (*n* = 36)	*P*	*n*	HR (95% CI)	*P*	*n*	HR (95% CI)	*P*
THBS2	2.49	3.29	**0.009**	179	1.42 (1.21–1.68)	**2.91E-05**	179	1.25 (1.04–1.51)	**0.016**
TAGLN	1.93	2.59	**0.034**	179	1.36 (1.16–1.58)	**9.87E-05**	179	1.21 (1.01–1.45)	**0.043**
DCN	1.7	2.16	0.168	179	1.24 (1.07–1.44)	**0.005**	179	1.05 (0.88–1.26)	0.584
PLA2R1	−1.96	−1.69	0.208	179	1.33 (1.06–1.67)	**0.015**	179	1.28 (0.97–1.69)	0.082
ERBB2	1.18	−0.06	**0.003**	233	0.87 (0.78–0.98)	**0.019**	224	0.99 (0.87–1.13)	0.921
ANXA10	−1.28	−2.38	0.034	233	0.95 (0.89–1.01)	0.107			
SATB2	−0.28	0.22	**0.007**	233	1.12 (0.89–1.40)	0.34			
PD-L1 (CD274)	−1.23	−1.06	0.627	233	1.07 (0.93–1.24)	0.341			
CK20 (KRT20)	−0.45	−1.31	0.019	233	1.03 (0.97–1.10)	0.354			
VSIG1	−0.13	−0.84	0.329	233	0.97 (0.91–1.04)	0.455			
MUC2	1.3	1.17	0.829	233	1.03 (0.95–0.46)	0.458			
PGC	0.13	0.36	0.782	233	0.98 (0.93–1.03)	0.5			
MUC16	−2.78	−1.77	0.130	233	1.02 (0.96–1.08)	0.567			
CLDN18	−1.79	−2.24	0.403	233	0.98 (0.92–1.05)	0.649			
MEP1A	0.07	−0.05	0.851	233	0.98 (0.91–1.06)	0.69			
GKN1	−0.06	0.06	0.294	233	1.07 (0.73–1.57)	0.721			
TYMS	0.1	0.02	0.658	233	1.04 (0.81–1.34)	0.746			
MUC5AC	0.44	−0.07	0.461	233	1.00 (0.94–1.07)	0.941			
PD-1 (PDCD1)	−1.25	−0.82	0.138	233	1.01 (0.86–1.17)	0.943			

Note: Difference in expression between stage groups Student *t* test; HR, hazard ratio, 95% confidence interval; multivariable analysis adjusted for age and stage, stratified by study site.

Survival was also assessed in upper and lower GI patients. Increased expression of *MUC2* was associated with better OS in lower GI tumors adjusted for age, stage, tumor type (colon/appendix), and stratified by study site (HR 0.72; 0.55–0.95; *P* = 0.020; Supplementary Table S5). There were no prognostic associations between gene expression and OS in upper GI tumors in multivariable analyses.

### Diagnosis

#### Clustering and diagnostic predictions

**Figure 3.**
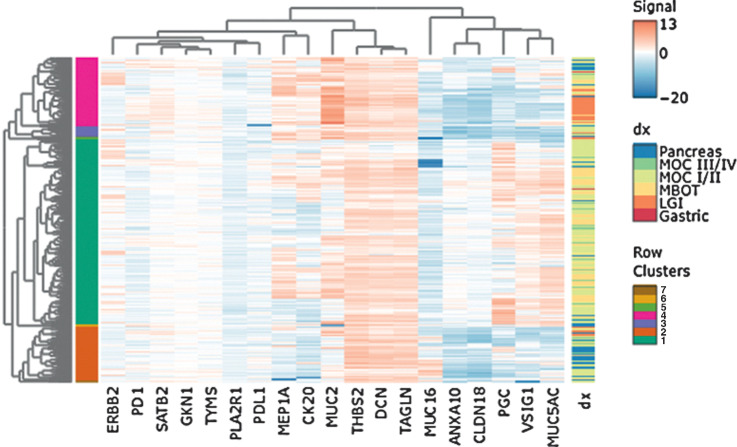
Heat map of unsupervised clustering analysis. Contains all samples with a concordant pathology diagnosis (*n* = 497), with MOC grouped by FIGO stage. Labels show main clusters and diagnoses. Gene-expression values are normalized and logarithm base 2 transformed. dx, diagnosis.

We identified nine genes in the RNA-seq analysis (Supplementary Methods, Fig. A) with the goal of differentiating between MBOT, MOC, and upper and lower GI cancers. A random forest model of these genes was trained and tested after stratified bootstrapping to produce balanced proportions in each diagnostic group class (Supplementary Table S6). We then used unsupervised hierarchical clustering to visualize clusters. To replicate the discovery analysis, we only included tissue samples from the ovary (MBOT, MOC, and upper and lower GI metastases to the ovary, *n* = 397). The mean testing error rate was 0.38 (Supplementary Fig. S6; Supplementary Table S7), and this poor validation is also reflected in the heat map (Supplementary Fig. S7). Following this, we ran a model with all 19 candidate genes and all pathology-concordant samples with stage data for MOC (*n* = 479). The mean testing error rate of the model was 0.33 (equivalent to an overall accuracy of 67%; Supplementary Fig. S6 and Supplementary Table S7). Lower GI samples were most accurately predicted (9/12, 75%), and upper GI samples were no greater than chance (50%). A heat map of these samples shows the lower GI samples clustering out in one main group, the pancreatic samples mainly in cluster 2, and the five gastric samples across clusters 1 and 4 along with MOC samples (Fig. 3).

### Comparison of gene expression across tumor groups

To examine similarities and differences between MOC, upper GI, and lower GI cancers, we compared gene expression between all pathology-concordant, invasive cases (*n* = 363). Although the random forest models and clustering showed that this gene set had a limited ability to distinguish tumor groups overall, the mean expression of several individual genes differed significantly between tumor groups (Supplementary Fig. S8). Sixteen genes significantly differed between MOC and lower GI tumors. *ANXA10, CLDN18, ERBB2/HER2, MUC16, MUC5AC, PGC*, and *VSIG1* showed significantly higher expression in MOC, and *MEP1A*, *PD1*, *DCN*, *TAGLN*, *THBS2*, *GKN1*, *CK20*, *MUC2*, and *SATB2* were significantly lower in MOC. Twelve genes differed significantly between MOC and upper GI. Two genes contrasted with opposing directions—*MEP1A* higher in MOC compared with upper GI, *MUC16* higher in MOC compared with lower GI. Expression of the immune marker *PD-1* was lower in MOC compared with both upper and lower GI (Supplementary Fig. S9) and *PD-L1* was relatively similar across all groups, but slightly lower in MOC compared with upper GI (*P* = 0.03).

### ERBB2/HER2 expression and implications for therapy

We analyzed mRNA expression of ERBB2/HER2 by NanoString for MBOT (*n* = 134), MOC (*n* = 243), lower GI (*n* = 55), and upper GI (*n* = 65). Expression of *ERBB2*/*HER2* was higher in MOC compared with both lower GI and upper GI and higher in stage I and II MOC, respectively, compared with MBOT (*P* < 0.001 and *P* < 0.001; Supplementary Fig. S10). A subset of cases (*n* = 37) was examined for *ERBB2*/*HER2* amplification using SISH, showing clear delineation between *ERBB2*/*HER2* amplified (*n* = 7) and nonamplified (*n* = 28, plus two equivocal) with respect to their mRNA expression levels (Supplementary Fig. S11A). We used these data to estimate that a threshold of normalized mRNA expression ≥2.5, represented potential amplification. When we applied this threshold from the SISH subset to all 243 MOC cases, 26% were considered high expressing, i.e., estimated to be amplified. The threshold was supported by the comparison of mRNA with protein expression from IHC, whereby 15 of 17 ERBB2/HER2 3+ staining tumors had >2.5 mRNA expression (Supplementary Fig. S11B). The proportion of high expression/amplified was higher in the low stages of MOC compared with the advanced stage (stage I: 36/139, 26%; stage II: 6/16, 38%; versus stage III/IV: 4/31, 13%; Supplementary Table S8). We did not observe differences by grade; however, *ERBB2*/*HER2* high/amplified were more common in MOC with an expansile pattern of invasion (chi-square *P* = 0.008), 50/163 (31%) compared with 4/39 (10%) of infiltrative cases. In summary, of the MOC cases with known poor prognostic features, ERBB2/HER2 high amplification was present in just 8 of 243 (3%). These were either infiltrative (4/39, 10%) or stage III/IV (4/31, 13%). There was no association between high expression/amplified *ERBB2*/*HER2* and OS (log-rank *P* = 0.2; Supplementary Fig. S12).

### Prediction modeling of nonrepresentative tissue

We trained and tested models using 246 concordant MOC and 139 concordant MBOT samples, to predict the diagnosis of 90 discordant samples that were submitted as carcinoma (MOC), but pathology review deemed MBOT. The random forest model had a relatively low mean testing error rate of 0.18 (Supplementary Fig. S6), and out of the 90 discordant cases, 53 were predicted to be MBOT, i.e., 59% of predictions were concordant with the pathologist review, and the rest were predicted to be MOC (Supplementary Table S9).

### Paired sample analysis

There were 33 pairs of samples from the same patient and the same diagnostic episode, consisting of 7 cases with MBOT and MOC, 1 case benign and MBOT blocks, 1 case left and right ovary blocks, 16 cases with two MBOT blocks, 2 cases with primary appendix and metastases to the ovary, and 6 cases with different metastatic tissue sites (Supplementary Table S10). We examined the correlation in gene expression between samples, and from 16 sets of MBOT tissue from different blocks for the same patient, the correlation was variable: 7 sets *R* > 0.9, 4 sets *R* = 0.7–0.9, and 5 sets <0.7. Two sets of primary low-grade appendiceal mucinous neoplasm (LAMN) and metastases to the ovary had very high correlation, *R* = 0.94 and *R* = 0.95. Three of 7 sets of MBOT and MOC from the same patient had a strong correlation (*R* > 0.9), 3 moderate *R* = 0.7–0.9, and 1 with poor correlation *R* = 0.40. Differences in correlation across sets of tumor samples were not related to differences in cellularity between samples, with 82% (9/11) of pairs with *R* < 0.8 both having tumor cellularity of <60%, as did 86% (18/21) of pairs with *R* > 0.8 (χ^2^ test, *P* = 0.8).

## Discussion

We found that increased expression of two markers, thrombospondin 2 (*THBS2*) and transgelin (*TAGLN*), was associated with poorer OS in MOC after adjustment for age and tumor stage. Thrombospondin 2 (THBS2) is a glycoprotein with a role in tumor growth, angiogenesis, and metastases, with high expression found to be associated with poorer survival in colorectal cancer at the mRNA and protein level (33). In ovarian cancer, *THBS2* mRNA expression has been shown to be upregulated in more aggressive tumors (malignant compared with borderline), advanced stage, and high grade (34). There may be role variations in different tumor types as *THBS2* has been reported to be an inhibitor of angiogenesis in cervical cancer (35). The role of *THBS2* in prognosis may be driven by an interaction with the extracellular matrix, enabling tumor progression and metastases. Transgelin (*TAGLN*) is an actin-binding protein, expressed in smooth muscle cells. Multiple studies in colorectal, gastric, pancreas, non–small cell lung cancer have shown increased *TAGLN* expression is associated with migration, invasion, and poor survival (36–38); however, others have suggested it is a tumor suppressor in colorectal cancer (39). Both prognostic genes appear to be expressed in the stroma, with upregulation of *TAGLN* in gastric stromal carcinoma-associated fibroblasts (40), and increased expression of *THBS2* implicated in tumor progression and poor prognosis in pancreatic cancer, excreted by stromal fibroblasts (41). This apparent stromal localization could also explain the higher expression levels observed in the infiltrative MOC compared with the expansile and subsequent prognostic association. Indeed, *THBS2* and *TAGLN* expression was higher in the samples with low tumor cellularity, inferring at least some expression may be due to the higher stromal content of the samples (Supplementary Fig. S13). Expression of *TAGLN* has been implicated with *KRAS* signaling in promoting proliferation in pancreatic cancer (42), *KRAS* mutations being the most frequent aberration in MOC (24). When both genes were combined in the same survival model, the associations were no longer significant, and the correlation was high (*R* = 0.8; Supplementary Table S11), suggesting a possible contributory effect of the two genes. Examination of the role of both *THBS2* and *TAGLN* in large clinical cohorts is critical, and validation of the current finding is needed to confirm the prognostic potential of these markers and to further explore their role in the biology of MOC.

We observed a time-dependent association between the pattern of invasion and OS, with an infiltrative pattern associated with poorer OS within 2 years from diagnosis, but not significant after 2 years. This finding was consistent when assessing FIGO stage I cases alone. This subset is arguably the most clinically relevant for the prognostic value of pattern of invasion: a poor outcome marker will influence decision-making when considering adjuvant chemotherapy or more vigilant monitoring for recurrence. Prior studies have reported varying outcomes with regard to progression-free survival (PFS) and OS in the infiltrative subtype (15–20); however, most have not adjusted survival models for age and stage, and no single study has observed a prognostic association in stage I MOC alone. The largest series to date (16) included 67 patients, and no multivariable analyses were performed. A similarly sized study of stage I only MOC (*n* = 64; ref. 17) found no statistically significant difference in PFS (*P* = 0.49) or OS (*P* = 0.18). Hada and colleagues reported that an infiltrative pattern was associated with poorer PFS (HR, 9.01; 2.28–61.41; *P* < 0.01) and OS (HR, 17.56; 2.58–393.24; *P* < 0.01), but this study was underpowered to analyze stage I alone. Combining stages I and II in univariate analysis (*n* = 38), they found a significant impact on PFS (*P* = 0.03), but OS was not evaluable (20). Time-dependent associations of prognostic factors have been described in other cancers such as triple-negative breast cancer (43), similar to our observation in patients with stage I MOC with an infiltrative pattern with early recurrence and death, and a low risk beyond the 2-year mark. The proportion of infiltrative cases here is lower than in many series, and it is possible that others used a different threshold or stringency in excluding metastases or heterogeneity between blocks has led to this difference. We classified an infiltrative pattern at >5 mm, and cases with only a small focus on destructive invasion were grouped with the expansile. Tabrizi and colleagues report a similar low frequency at 13% (4/31) in a population-based series and suggest that other institutional studies with higher rates of infiltrative cases may reflect more complex, selected populations (19). Of note in the current study, four of the stage I cases were reported to have an anaplastic component: two infiltrative and one expansile, all of whom died within 2 years, and one infiltrative case was alive after 7 years. Although anaplastic carcinoma arising in mural nodules is considered to infer a more aggressive disease, some report that their presence in stage I disease does not influence outcome (18). It cannot be ruled out that the small number of anaplastic carcinoma cases in this study influenced our findings. Given that infiltrative invasion is a feature of metastatic neoplasms to the ovary and was observed more frequently in higher stage MOC, we also cannot rule out that some of these cases represent undiagnosed metastases from a different primary site or inadequate staging of disease. Expression of the two prognostic markers *THBS2* and *TAGLN* was significantly higher in the infiltrative subtype compared with expansile. In contrast, *ERRB2* encoding HER2 expression was lower in infiltrative MOC compared with expansile MOC, which is consistent with other reports of HER2-positive MOC on IHC associated with the expansile subtype and better prognosis (44).

This study has replicated the survival patterns seen in the literature for MOC (6) and GI tumors, showing that advanced-stage MOC and upper GI cancers have significantly poorer survival than MBOT, stage I MOC, and lower GI cancers. Notably, the difference observed in 5-year survival between stage I (79%) and II (69%) indicates that studies in MOC should not combine these “low” stages together in analyses, which is the practice for ovarian endometrioid carcinomas (45).

The discovery RNA-seq analysis identified a 9-gene classifier to help differentiate between MBOT, MOC, and metastases to the ovary; however, we did not validate this in the larger cohort. This could be due to cohort differences, such as inclusion of benign and “seromucinous” cases and few GI tumors in the RNA-seq data set. We were limited by the 19-gene panel in this large follow-up study using formalin-fixed paraffin-embedded tissue, and more work is needed to identify other possible diagnostic classifiers that may have been missed by this study. Despite this, clustering of the whole gene set found that most lower GI tumors separated out prominently in one main cluster, but MOCs and upper GI grouped together. This, along with differences in expression between groups, revealed more differences between MOC and lower GI compared with MOC and upper GI. Recent improvements in diagnostic classification now include the use of CK7 and SATB2 for lower GI metastases (4); in contrast, differential markers for upper GI tumors remain elusive. One prior study showed potential for *MEP1A* with lower membranous staining in MOC compared with pancreatic cancers (46); however, in our cohort, the mean mRNA expression was higher in MOC compared with upper GI tumors (Supplementary Fig. S8), including in comparison with pancreatic tumors alone (*P* = 0.006; Supplementary Fig. S8). It is possible that the challenges of differentiating MOC from mucinous pancreatic and gastric cancers could shift the therapeutic strategies for MOC. Considering the similarities between MOC and pancreatic tumors, we see high rates of coexisting *CDK2NA* inactivation (76%), and a similar frequency of *TP53* mutations (∼60%; ref. 24). Likewise, the gastroesophageal junction tumors share the features of *ERBB2* amplification and *TP53* mutations (47). There is an argument to shift focus from trying to seek differences between groups and look at opportunities for basket-style clinical trials of either systemic or targeted therapies by including advanced-stage MOC together with GI cancers based on shared molecular alterations (48). For example, FOLFIRINOX is the standard of care in metastatic pancreatic cancers but has not been investigated in advanced-stage MOC (49). In addition, 20% to 30% of MOCs have been reported to harbor *ERBB2* amplification (26, 27, 50), consistent with our finding (26%). Our findings on ERBB2/HER2 amplification/overexpression confirm the results of previous studies (26, 27, 44, 50), including the observation of a lower frequency of *ERBB2/HER2* high/amplified cases in advanced-stage MOC (4/31, 13%). Similarly, 4/39 (10%) of infiltrative subtype MOC were *ERBB2/HER2* high/amplified compared with 31% with an expansile pattern, consistent with the study by Kim and colleagues who reported 0/9 infiltrative and 14/37 expansile were HER2-positive (44). If the suitable population for anti-HER therapy were limited to high-stage or infiltrative MOC, our data suggest that approximately 3% of patients may be considered. Despite this, in addition to high/amplified cases, there have been promising developments in the treatment of HER2-low (IHC 1+) in breast cancer (51), which may broaden eligibility to these therapies for patients with advanced-stage MOC and HER2 1+ or 2+ on IHC. Additional important developments in anti-HER2 directed therapy in gastric cancer now include antibody–drug conjugates such as trastuzumab deruxtecan in the advanced setting (52), and a potential role for XELOX-T (oxaliplatin, capecitabine, and trastuzumab) in locally advanced, resectable gastric cancer (53). The latter therapy regimen is based on a small phase II study; however, future large randomized studies could arguably adopt a basket design to include *ERBB2/HER2*-amplified MOCs as well as potentially all tumors with high ERBB2/HER2 expression on IHC. Indeed, the current Bouquet-ENGOT-gyn2 rare ovarian cancers basket trial (ClinicalTrials.gov identifier: NCT04931342) includes an arm for *ERBB2/HER2*-amplified/mutated cases for treatment with trastuzumab emtansine.

The current study did not provide a simple mRNA profile that can be used diagnostically to distinguish MBOT from MOC, and it highlighted the heterogeneity through varying concordance of expression between borderline and invasive carcinoma and between multiple borderline tumor blocks from the same patient. Whether the 25% of MOC cases considered borderline on pathology review reflect a genuine discrepancy between pathologists, or the submission of a nonrepresentative block from a heterogeneous tumor remains unclear and should be the subject of further studies. Interestingly, a recently reported French cohort (*n* = 79) with access to all blocks or a minimum of 5 blocks also reclassified 18% of MOC as MBOT (16).

In the context of exploring better therapeutic options for MOC, we observed lower levels of expression of *PD-1* in comparison with GI tumors, and similar levels of *PD-L1*. As immunotherapy is now being investigated in multiple cancer types, further studies with appropriate IHC scoring for *PD-1* and *PD-L1* should be carried out to understand whether a subset of MOC may benefit from immunotherapy.

There are several limitations to the current study that has combined samples and data on a large scale over many years. Because tumor heterogeneity is well recognized in MOC, it is possible that the blocks sectioned for the study were not representative. This was highlighted by the discordant diagnoses which may be due to sampling or individual pathologist's interpretations. Although 30% of mucinous ovarian tumors (*n* = 104) had IHC performed for CK7, CK20, CDX2, SATB2, and PAX8 in a prior study (4), we were unable to perform this diagnostic panel on all cases and could not confirm whether they were done as part of routine pathologic assessment. Notwithstanding this limitation, the majority of misclassified samples related to discordance between MOC and MBOT. The IHC panel would be of limited assistance to differentiate these entities as the diagnosis is based on H&E. This panel would have limited utility in differentiating upper GI from MOC due to their similarities in the IHC phenotype. In addition, the lack of guidelines for HER2 scoring in MOC meant that we employed those used in serous endometrial cancer, and this could have misclassified some cases on IHC; however, the follow-up SISH to determine HER2 amplification would mitigate this. Survival analyses lacked residual disease, progression, and cause of death data. A major strength is that this is the largest series to date of gene-expression profiles of MOC and includes comparisons with upper and lower GI tumors on the same profiling platform. Future work could also assess mutation profiles to identify mRNA expression differences in *KRAS*/*TP53* mutant and wild-type subsets.

This analysis of a large series of mucinous ovarian carcinomas has identified two potential prognostic biomarkers in *THBS2* and *TAGLN*, which could have clinical utility and deserve further investigation. In addition, we confirmed the importance of an infiltrative pattern of invasion as a risk indicator for early recurrence and mortality. Given their rarity, there is a strong argument supporting the inclusion of MOC in basket trials with similar and much more common GI mucinous cancers.

## Supplementary Material

Supplementary Methods 1 Supplementary Methods

Supplementary Tables S1-S11 Supplementary Tables S1-S11

Supplementary Figures S1-S13 Supplementary Figures S1-S13
