# Supplementary material for: Gene-Expression Profiling of Mucinous Ovarian Tumors and Comparison with Upper and Lower Gastrointestinal Tumors Identifies Markers Associated with Adverse Outcomes
Source: Clin Cancer Res. 2022 Oct 7;28(24):5383–95. doi: 10.1158/1078-0432.CCR-22-1206 (PMC9751776; doi:10.1158/1078-0432.CCR-22-1206)
Supplement: Supplementary Methods 1 — Supplementary Methods [file ccr-22-1206_supplementary_methods_1_suppms1.docx]

Supplementary methods

**Gene selection from discovery datasets**

1. **NanoString 513 gene panel**

This study(1, 2) of predominantly high-grade serous ovarian cancer contained 60 Mucinous Ovarian Carcinomas (MOC) in the dataset for analysis of 513 genes (Appendix 1). We modelled the top genes that were either differentially expressed between low (I/II, n=49)) and high (III/IV, n=11) stage MOC (Table A) or associated with poorer overall survival (Table B).

Table A: Top 25 genes differentially expressed between low (I/II) and high (III/IV) stage MOC by Wilcoxon signed-rank text.

| GENE | p-value (un-adj) |
| --- | --- |
| TUBB6 | 6.34E-04 |
| TAGLN | 8.82E-04 |
| DCN | 2.39E-03 |
| KCNE3 | 3.66E-03 |
| THBS2 | 4.48E-03 |
| ITM2B | 6.23E-03 |
| PLA2R1 | 6.64E-03 |
| WT1 | 7.08E-03 |
| AXL | 7.54E-03 |
| NOTCH3 | 7.54E-03 |
| NTRK2 | 8.02E-03 |
| NUAK1 | 8.02E-03 |
| FBN1 | 8.53E-03 |
| LUM | 8.53E-03 |
| POSTN | 1.09E-02 |
| INHBA | 1.15E-02 |
| ATP8B4 | 1.22E-02 |
| FGFR1 | 1.22E-02 |
| IL6 | 1.22E-02 |
| EZR | 1.30E-02 |
| VSIG4 | 1.30E-02 |
| HGF | 1.46E-02 |
| KLF10 | 1.46E-02 |
| PAX8 | 1.54E-02 |

Table B: Associations between gene expression, stage and survival in the discovery dataset

|  | NanoString I discovery dataset | | | | | | |
| --- | --- | --- | --- | --- | --- | --- | --- |
|  | Mean expression by stage group | |  | Univariate (n=60) | | Multivariable (n=60) | |
|  | I/II (n=49) | III/IV (n=11) | p | HR (95% CI) | p | HR (95% CI) | p |
| THBS2 | -2.8 | -1.44 | **0.002** | 1.84 (1.26-2.70) | **0.002** | 1.13 (0.82-1.57) | 0.454 |
| TAGLN | -0.57 | 0.74 | **0.001** | 1.73 (1.23-2.45) | **0.002** | 1.15 (0.76-1.74) | 0.497 |
| DCN | -1.64 | -0.21 | **0.001** | 1.47 (1.02-2.12) | **0.037** | 0.92 (0.63-1.34) | 0.672 |
| PLA2R1 | -6.69 | -5.81 | **0.004** | 1.34 (0.88-2.05) | 0.178 |  |  |

1. **RNASeq analysis**

RNAseq data from the Genomic Analysis of Mucinous Tumours (GAMuT) cohort(3) (<https://ega-archive.org/datasets/EGAD00001005190>) was used for exploratory RNAseq analysis to identify class specific genes. The dataset included 80 patient samples: 11 mucinous benign tumors, 19 mucinous borderline ovarian tumors (MBOT), 40 MOC, and 10 metastases to the ovary from different primary sites. Reads were aligned to a SNP and transcript aware index of GRCh38 with HISAT2.(4) Gene counts were generated with HTseq-count (5) using the Gencode v24 annotation. Samples with less than 10M reads mapping to exons were excluded. Genes where the average CPM was less than 0.5 and no sample had a log2CPM value >7.0 were excluded. Clustering was performed on log2CPM counts with TMM normalization.(6) The log2CPM was stabilised with differing prior count values ranging from 0.5 (standard value used in differential expression) to 30. Increasing the prior count value in the log2CPM calculation reduces the variance of lowly expressed genes and decreases their weight in clustering. Clustering was performed using v11.2 of the seaborn python package,(7) across a range of prior count values, and high variance gene rank cut offs. Selected genes were robust across the complete average weighted centroid median and ward clustering methods and range of values explored (data not shown). A log2CPM prior count of 8, ward clustering and high variance gene cut off of 50 provided the best recapitulation of prior histology category. Unsupervised hierarchical clustering of samples was performed to identify biologically distinct groups. Genes were selected representing each cluster group, manually interpreting the clusters of samples as enriched for benign, borderline, MOC, MOC-MUC16+ and high-grade MOC/non-ovarian. Final manual selection included genes that had high expression, performed consistently across different clustering methods and parameter settings, were biologically plausible, and had available antibodies for future clinical utility. These 9 classifier genes were *MUC16* (encoding CA125), *GKN1*, *PGC*, *MEP1A*, *KRT20* (encoding CK20), *MUC5AC*, *CLDN18*, *VSIG1* and *ANXA10*.

Figure A: Heatmap of 9 classifier genes from RNASeq discovery analysis


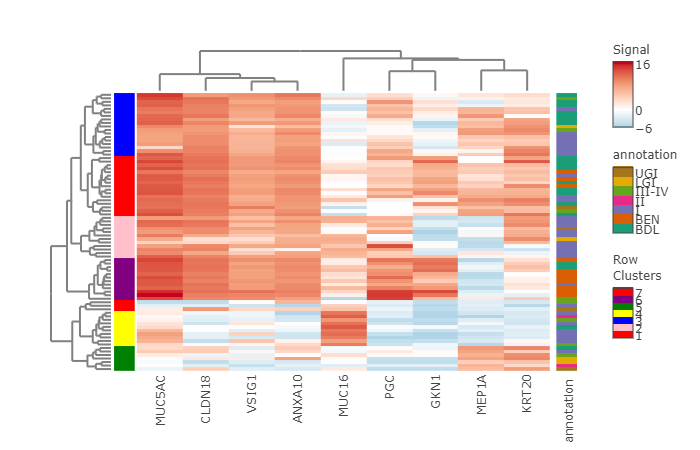


1. **NanoString plexset housekeeping gene selection**

The aim was to select 5 housekeeping (HK) genes for internal normalisation of the NanoString data. The HK genes need to include a range of low, medium and high expressing, with limited variation in expression across the ovarian and gastrointestinal tumours included in the validation cohort. Two datasets were used:

Dataset 1: Publicly available TCGA RNASeq data (https://portal.gdc.cancer.gov/), for the following tumour groups: colorectal adenocarcinomas (COAD), ovarian (OV), pancreas (PAAD), stomach (STAD).

Dataset 2: The Genomic Analysis of Mucinous Tumours (GAMuT) cohort RNASeq data (Gorringe lab, Peter MacCallum Cancer Centre). One of the limitations of Dataset 1 was that the OV cases are predominantly high-grade serous cancers which we expect to have a different gene expression profile to those with mucinous histology. The GAMuT dataset contains 6 different tumour groups: mucinous ovarian carcinoma (MOC), gastrointestinal metastases (EOM-GIT), mucinous borderline tumour (BL-MOT), benign mucinous tumour (BEN-MOT), endometrial metastases (EOM-Endo), metastases, unknown primary (MET).

We calculated the median expression value (in FPKM) for each gene within each tumor group (Figure B) and grouped genes into four groups based on their 25% quantiles in FPKM values to obtain low, medium, medium-high and high expressing genes.

To assess the stability across tumor groups, we took the log10(FPKM) and performed an ANOVA to compare between tumor groups, with high p-values representing no difference in expression between groups.


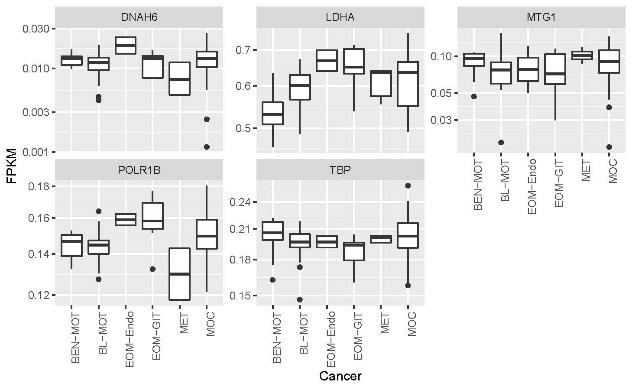
Figure B: Expression of selected housekeeping genes across TCGA (left) and RNASeq (right) datasets


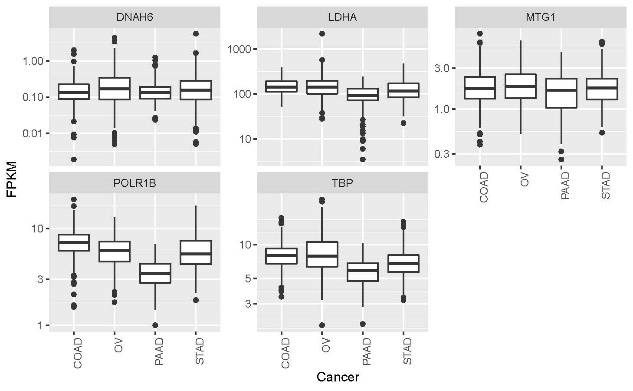


Appendix 1: Full gene list from NanoString discovery dataset

| AADAC | BECLIN1 | COL11A1 | ERBB2 | GSTP1 | KGFLP2 | MRPL45 | PGRA | RPL21 | SUPT6H | WWP1 |
| --- | --- | --- | --- | --- | --- | --- | --- | --- | --- | --- |
| ABCA1 | BIRC5 | COL1A2 | ERBB3 | GTF2H5 | KIAA1033 | MRPS27 | PGRB | RPL23 | SVIL | XRN2 |
| ABCB1 | BMS1 | COL3A1 | ERBB4 | GTPBP3 | KIF1A | MS4A3 | PHF20 | RPL41 | TAGLN | YWHAB |
| ABCC2 | BNIP3 | COL4A6 | ERGIC3 | GUSB | KIF24 | MSH4 | PHKG2 | RPS23 | TAP1 | ZBED1 |
| ABCC3 | BNIP3L | COL5A1 | ESD | HBB | KIF3B | MTA1 | PI3 | RSPH1 | TBC1D8B | ZC3H13 |
| ABCC4 | BOP1 | COL5A2 | ESR1 | HGF | KIF4A | MUC5B | PIGH | RSPO1 | TBP | ZFHX4 |
| ABCC5 | BRAF | CPNE1 | ESR2 | HHAT | KIFC1 | MVB12A | PIK3CA | S100A6 | TBX2 | ZNF12 |
| ABCE1 | BRCA1 | CPNE8 | EZR | HIF1A | KIT | MYC | PJA2 | SAC3D1 | TCF7L1 | ZNF165 |
| ADAM12 | BRCA2 | CRABP2 | FABP4 | HINT2 | KLF10 | MYCL1 | PKM1 | SACS | TDRD3 | ZNF423 |
| ADAMDEC1 | BYSL | CRISPLD2 | FAM126B | HIST1H2AM | KLHL7 | MYCN | PKM2 | SALL2 | TESK1 | ZNF668 |
| ADCYAP1R1 | C10orf116 | CSF1R | FAM58A | HIST1H2BD | KLK7 | MyD88 | PLA2R1 | SCGB1D2 | TFF1 | ZNF830 |
| ADH1B | C10orf82 | CSNK1G3 | FANCG | HIST1H2BE | KPNA2 | MYOD1 | PLAC4 | SCGB2A1 | TFF3 | ZNHIT2 |
| AFP | C19orf12 | CTHRC1 | FAP | HIST1H2BG | KRAS | NBEA | PLK2 | SEMA4D | TFPI | ZSCAN16 |
| AKT1 | C1orf173 | CTLA4 | FAS | HIST1H2BH | KRT13 | NBN | POLA2 | SEMA6A | TFPI2 |  |
| AKT1S1 | C7orf26 | CTNNB1 | FBN1 | HIST1H2BI | KRT6 | NF1 | POLR1D | SENP8 | TGFBR2 |  |
| AKT2 | CAMK1 | CTNNBL1 | FCER1G | HMGA2 | LBP | NF2 | POLR2H | SERPINA5 | THBS2 |  |
| ALG10B | CAPN2 | CTSD | FEN1 | HMGB3 | LDHB | NOTCH1 | POSTN | SERPINE1 | TIGAR |  |
| ALG8 | CASP8 | CTSK | FGF1 | HNF1A | LGALS14 | NOTCH3 | PPL | SHARPIN | TIMP3 |  |
| ANKRA2 | CAT | CX3CR1 | FGFR1 | HNF1B | LGALS4 | NPEPL1 | PPP2R4 | SHPRH | TIPARP |  |
| ANKRD1 | CAV1 | CXCL10 | FGFR3 | HOXA9 | LGR6 | NRAS | PRDX1 | SIRT5 | TLR4 |  |
| ANXA4 | CCDC171 | CXCL11 | FGFR4 | HOXD9 | LIN28B | NRIP1 | PRTFDC1 | SLAMF7 | TMCO3 |  |
| AOX1 | CCL5 | CXCL14 | FHIT | HPRT1 | LOC81691 | NTN2L | PSIP1 | SLAMF8 | TMEM45A |  |
| AP3S1 | CCND1 | CXCL17 | FKSG2 | HRAS | LOX | NTRK1 | PSME2 | SLC12A3 | TOMM20 |  |
| APBB2 | CCNE1 | CXCL9 | FLT1 | HSF1 | LPAR3 | NTRK2 | PSRC1 | SLC16A1 | TOP1 |  |
| APC | CD2 | CYB561 | FLT4 | HSP90AA1 | LRCH1 | NUAK1 | PTCH1 | SLC16A4 | TP53 |  |
| APC2 | CD27 | CYP2C18 | FMN2 | HYOU1 | LRFN4 | NUAK2 | PTEN | SLC25A19 | TPT1 |  |
| APPL2 | CD302 | CYP4B1 | FN1 | IDO1 | LRRC15 | NUCB2 | PTGER3 | SLC25A30 | TPX2 |  |
| AR | CD38 | CYTIP | FNDC3A | IFI44 | LRRC50 | NUP85 | PTGS1 | SLC2A3 | TRIL |  |
| ARHGEF11 | CD3D | DAB2 | FOLR1 | IFT88 | LUM | OASL | PTGS2 | SLC37A4 | TRIM27 |  |
| ARID1B | CD3e | DCN | FOXC2 | IGF1 | MAB21L1 | OLFML3 | PTH2R | SLC39A10 | TRIT1 |  |
| ARID5B | CD47 | DHRS1 | FOXJ1 | IGF1R | MAD1L1 | OPA1 | PTPN11 | SLC3A1 | TSC1 |  |
| ASB7 | CD55 | DHRS7 | FOXP3 | IGF2 | MAK | OR1G1 | PTPRT | SLC40A1 | TSC2 |  |
| ASRGL1 | CD68 | DHX35 | FOXRED2 | IGFBP1 | MAL | OR51V1 | QPRT | SMARCA4 | TSHR |  |
| ATG16L1 | CD74 | DIS3 | FRAP1 | IGFBP2 | MANBAL | OR52E4 | RAC1 | SMARCB1 | TSNAXIP1 |  |
| ATM | CD8A | DKK4 | FUT3 | IGFBP4 | MAP1LC3A | OR52N4 | RAD50 | SMO | TSPAN8 |  |
| ATP5A1 | CDC42 | DNAI1 | GAD1 | IGHM | MAP2K4 | PARP4 | RAD51B | SNRPA1 | TTC39B not P2 |  |
| ATP5G3 | CDC7 | DNAJC9 | GALNT6 | IGJ | MAP3K11 | PAX2 | RAD51C | SORBS3 | TTC39B P2 |  |
| ATP5H | CDCA8 | DPAGT1 | GAPDH | IGKC | MAP4K3 | PAX8 | RALA | SORL1 | TUBB6 |  |
| ATP8B4 | CDH1 | DUSP1 | GCNT3 | IL22 | MBNL2 | PBX1 | RALB | SORT1 | UCA1 |  |
| AURKA | CDK12 | DUSP4 | GFPT2 | IL6 | MCM3 | PCDH9 | RALGAPB | SOX10 | UCHL1 |  |
| AURKB | CDK4 | E2F1 | GFRA1 | INHBA | MDM2 | PCK2 | RARRES1 | SOX17 | UCP2 |  |
| AXL | CDK6 | E2F6 | GJB1 | ISG15 | MDM4 | PD-1 | RASA1 | SPARC | UQCC |  |
| B4GALT5 | CDK8 | EGFL6 | GK5 | ITM2B | MEOX2 | PDE6D | RB1 | SPTLC2 | USP8 |  |
| BAALC | CDK9 | EGFR | GLI2 | ITPKC | MEST | PDGFRA | RBMS3 | SQSTM1 | UTP14C |  |
| BABAM1 | CDKN2A | ENOX1 | GLUT1 | JAG2 | MET | PDGFRB | RCBTB1 | SRI | VANGL1 |  |
| BAP1 | CDKN3 | ENPP1 | GMNN | JAK2 | MINPP1 | PD-L1 | RHOBTB3 | STAT5B | VCAN |  |
| BAX | CEACAM5 | ENTPD1 | GMPR | KCNE3 | MITF | PDS5B | RICTOR | STATH | VSIG4 |  |
| BCAP31 | CHCHD8 | EPAS1 | GNA12 | KCNMB3 | MPZL2 | PDZK1IP1 | RNASE2 | STC1 | WDR91 |  |
| BCHE | CLUH | EPB41L3 | GNG4 | KDM5D | MRE11A | PEX3 | RNASEL | STK16 | WRB |  |
| BCL2 | COBL | EPCAM | GPR64 | KDR | MROH5 | PGGT1B | RNF114 | STX17 | WT1 |  |

**References**

1. Millstein J, Budden T, Goode EL, Anglesio MS, Talhouk A, Intermaggio MP, et al. Prognostic gene expression signature for high-grade serous ovarian cancer. Annals of Oncology. 2020;31(9):1240-50.

2. Talhouk A, George J, Wang C, Budden T, Tan TZ, Chiu DS, et al. Development and Validation of the Gene Expression Predictor of High-grade Serous Ovarian Carcinoma Molecular SubTYPE (PrOTYPE). Clin Cancer Res. 2020;26(20):5411-23.

3. Cheasley D, Wakefield MJ, Ryland GL, Allan PE, Alsop K, Amarasinghe KC, et al. The molecular origin and taxonomy of mucinous ovarian carcinoma. Nature Communications. 2019;10(1):3935.

4. Kim D, Paggi JM, Park C, Bennett C, Salzberg SL. Graph-based genome alignment and genotyping with HISAT2 and HISAT-genotype. Nature Biotechnology. 2019;37(8):907-15.

5. Anders S, Pyl PT, Huber W. HTSeq—a Python framework to work with high-throughput sequencing data. Bioinformatics. 2014;31(2):166-9.

6. Robinson MD, Oshlack A. A scaling normalization method for differential expression analysis of RNA-seq data. Genome Biology. 2010;11(3):R25.

7. Waskom ML. seaborn: statistical data visualization. Journal of Open Source Software. 2021;6(60):3021.
