# Supplementary material for: Gene-Expression Profiling of Mucinous Ovarian Tumors and Comparison with Upper and Lower Gastrointestinal Tumors Identifies Markers Associated with Adverse Outcomes
Source: Clin Cancer Res. 2022 Oct 7;28(24):5383–95. doi: 10.1158/1078-0432.CCR-22-1206 (PMC9751776; doi:10.1158/1078-0432.CCR-22-1206)

Figure S1: flowchart of sample inclusions and exclusions


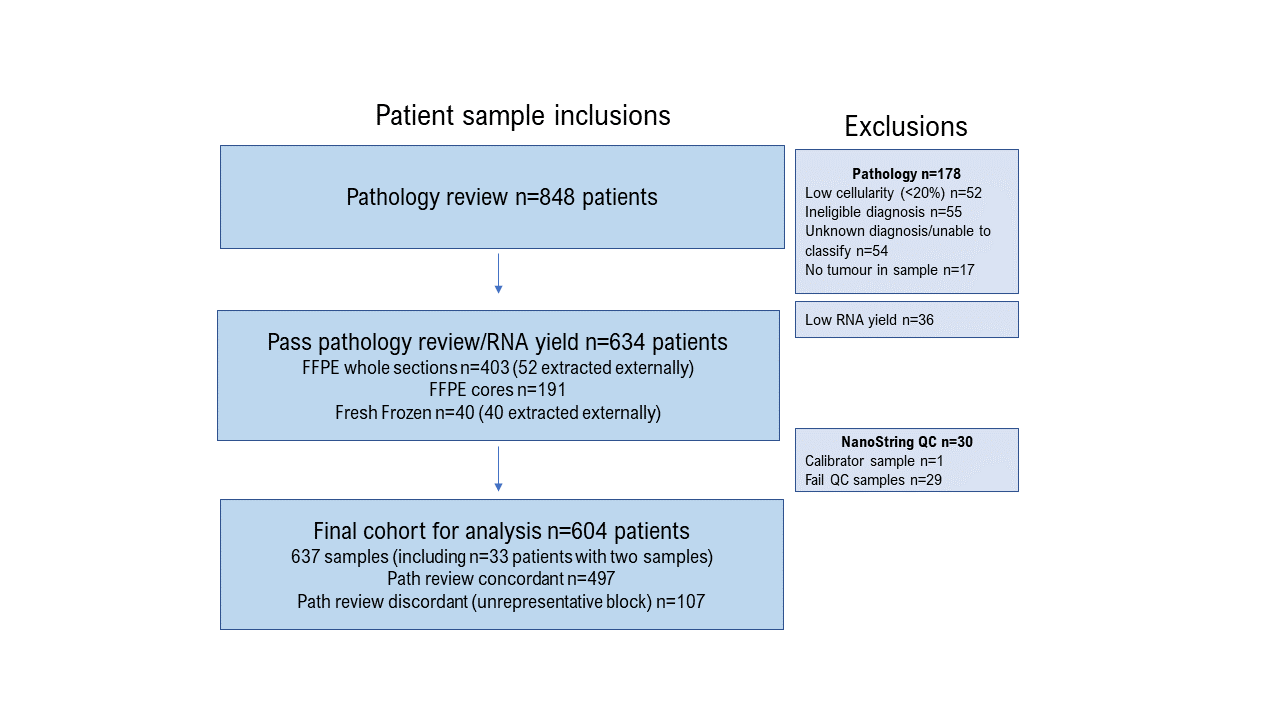


Figure S2: Proportion of MOC cases with expansile/infiltrative pattern of invasion by FIGO stage (n=208)

Figure S3: Comparison of gene expression between infiltrative and expansile MOC in all Stages and Stage 1 alone. Results of ANOVA with Tukey’s post-hoc test, p-values are adjusted for multiple comparisons.

| Gene | Difference in expression (all Stage MOC, n=208) | Difference in expression (Stage I MOC, n=134) |
| --- | --- | --- |
| DCN |  |  |
| TAGLN |  |  |
| THBS2 |  |  |
| GKN1 |  |  |
| MUC2 |  |  |
| SATB2 |  |  |
| PLA2R1 |  |  |
| ERBB2 |  |  |
| ANXA10 |  |  |
| CLDN18 |  |  |
| VSIG1 |  |  |
| PGC |  |  |
| PD1 |  |  |
| MUC16 |  |  |
| MUC5AC |  |  |
| CK20 |  |  |
| MEP1A |  |  |
| PDL1 |  |  |
| TYMS |  |  |

Figure S4: Kaplan Meier curves of overall survival of ‘high’, ‘medium’ and ‘low’ tertiles of mRNA expression of THSB2 (left) and TAGLN (right) in MOC (n=185)


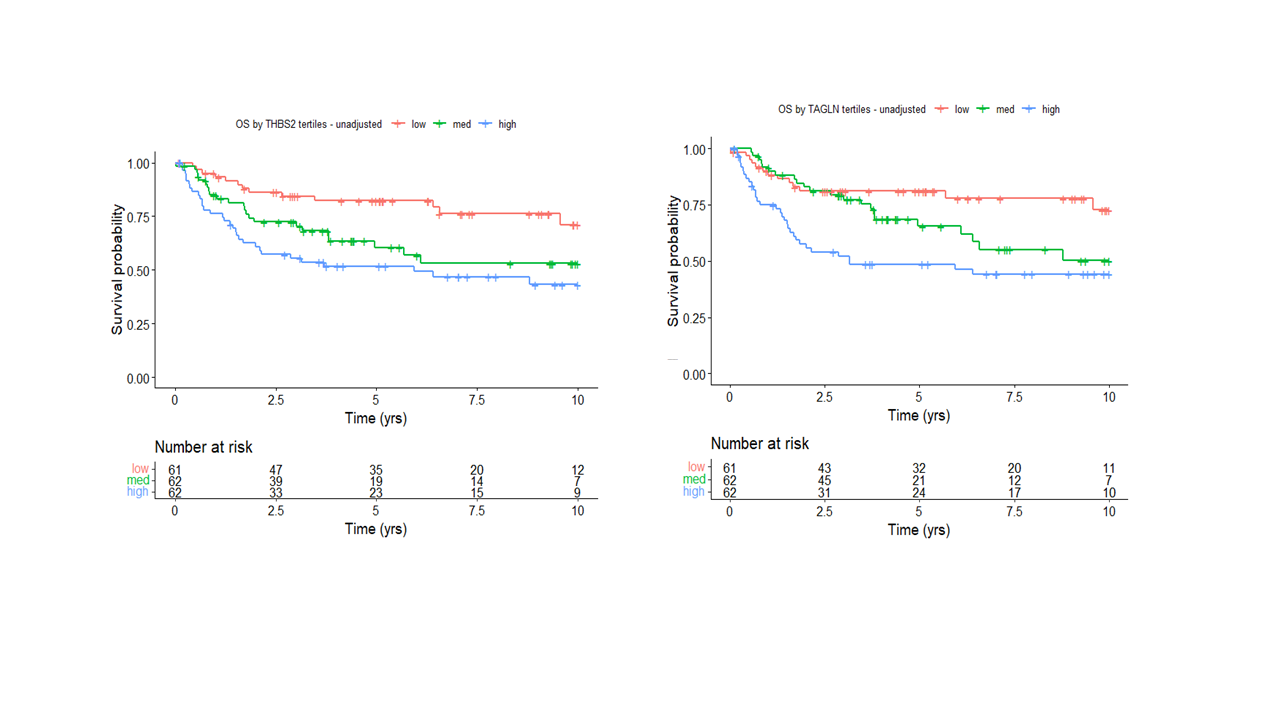


Figure S5: Boxplots of differential mRNA expression in prognostic genes THBS2 (left) and TAGLN (right) between expansile and infiltrative pattern of invasion in MOC (all stages, n=208).


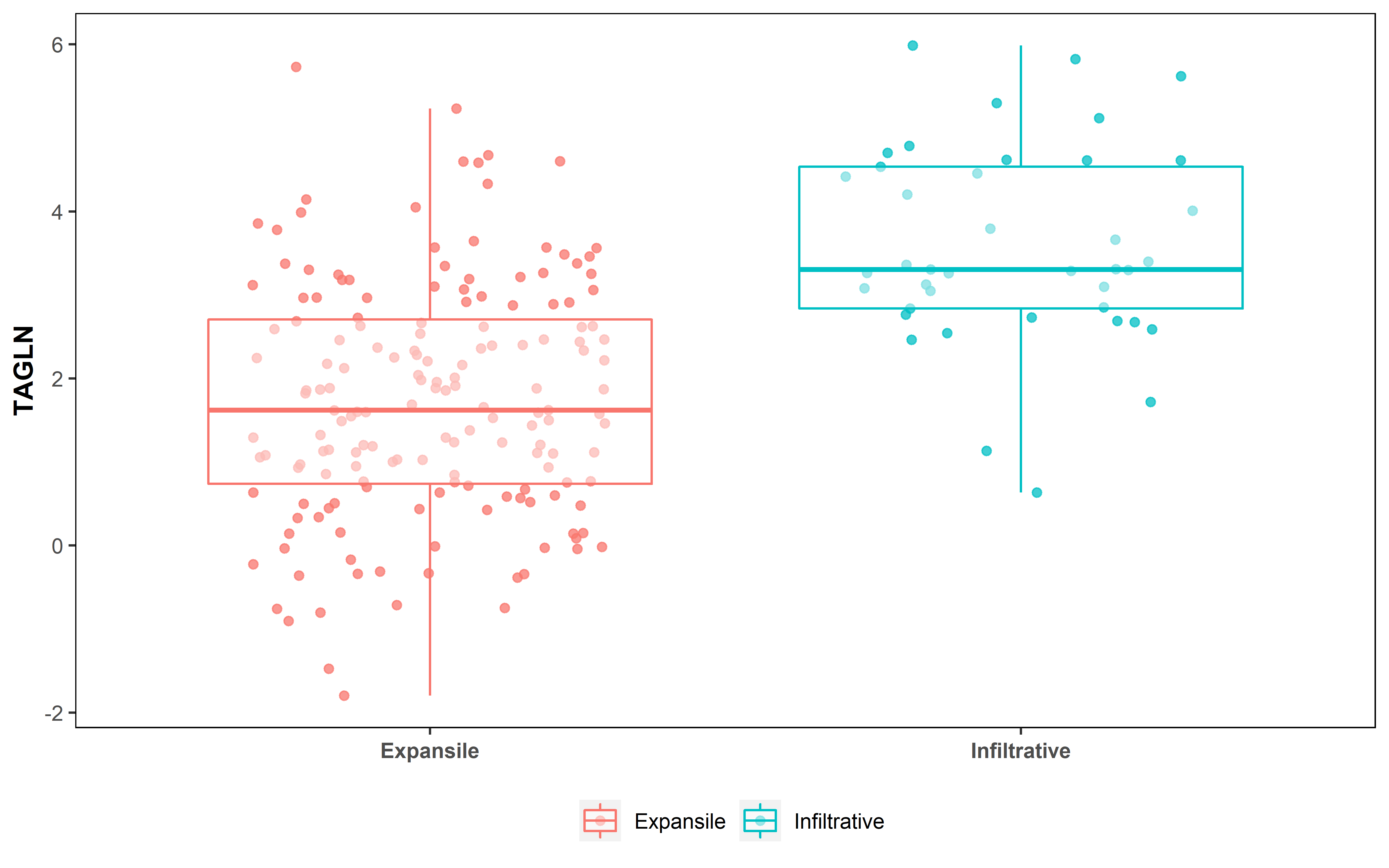

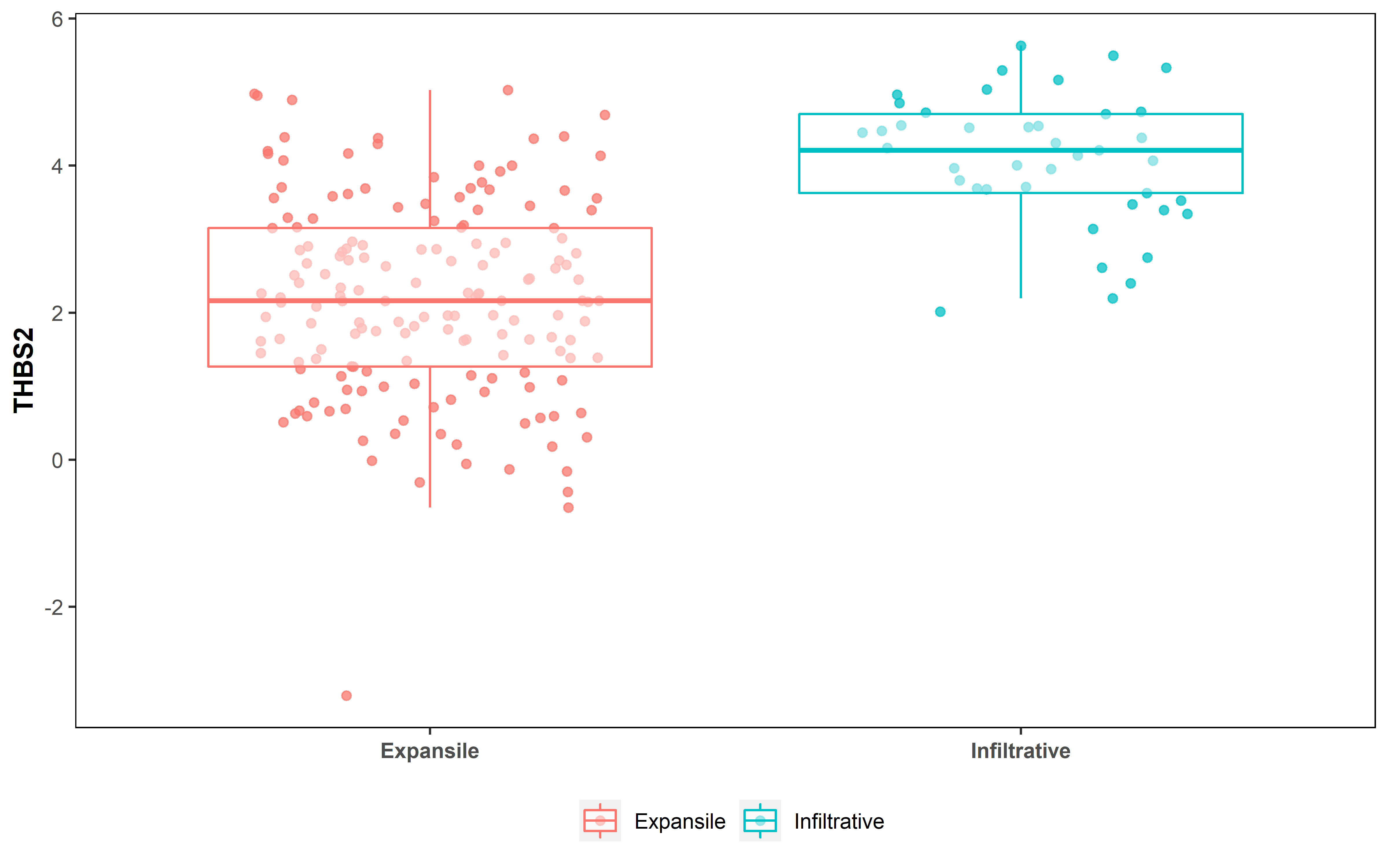


**p<0.001**

**p<0.001**

Figure S6: The mean error rates estimated using the testing set from 100 stratified bootstrapped random forest models.

**
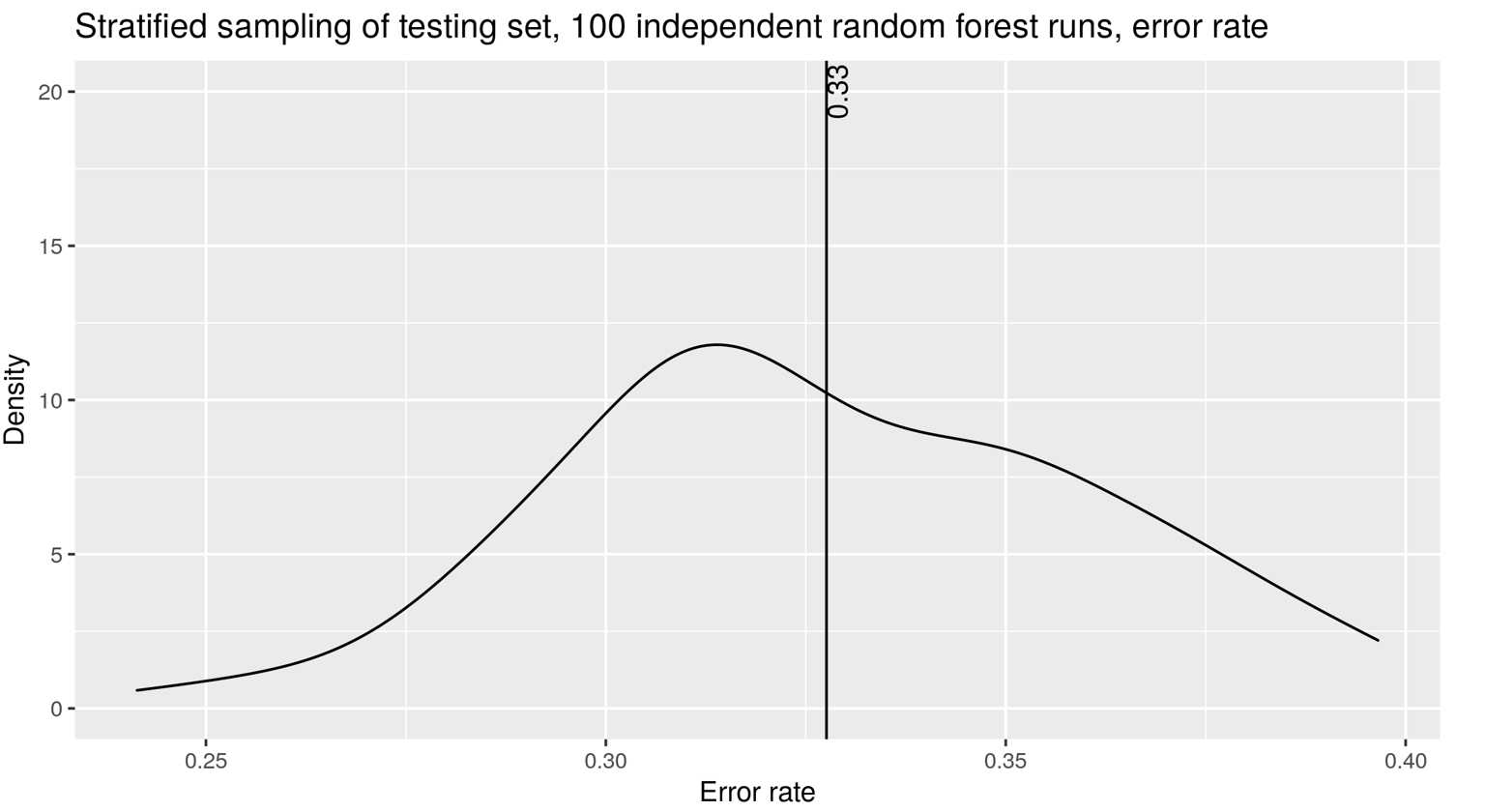
**A) diagnoses with all genes, all samples with a concordant diagnosis upon pathology review (n=497)

**
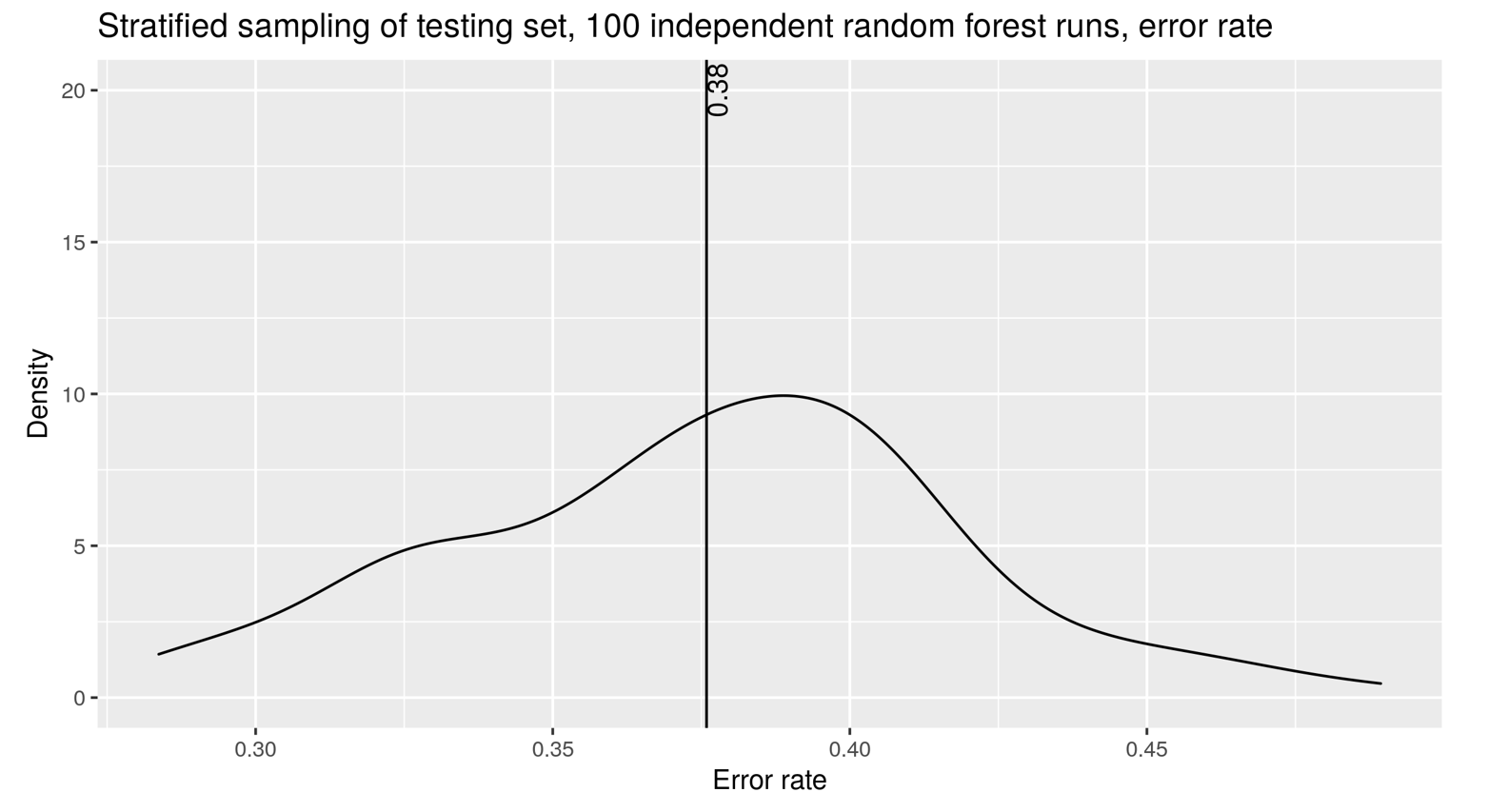
**B) diagnoses with 9 genes, a concordant diagnosis following pathology review and ovarian tissue samples only (n=397)

C) for classifying discordant cases into MBOT/MOC diagnoses


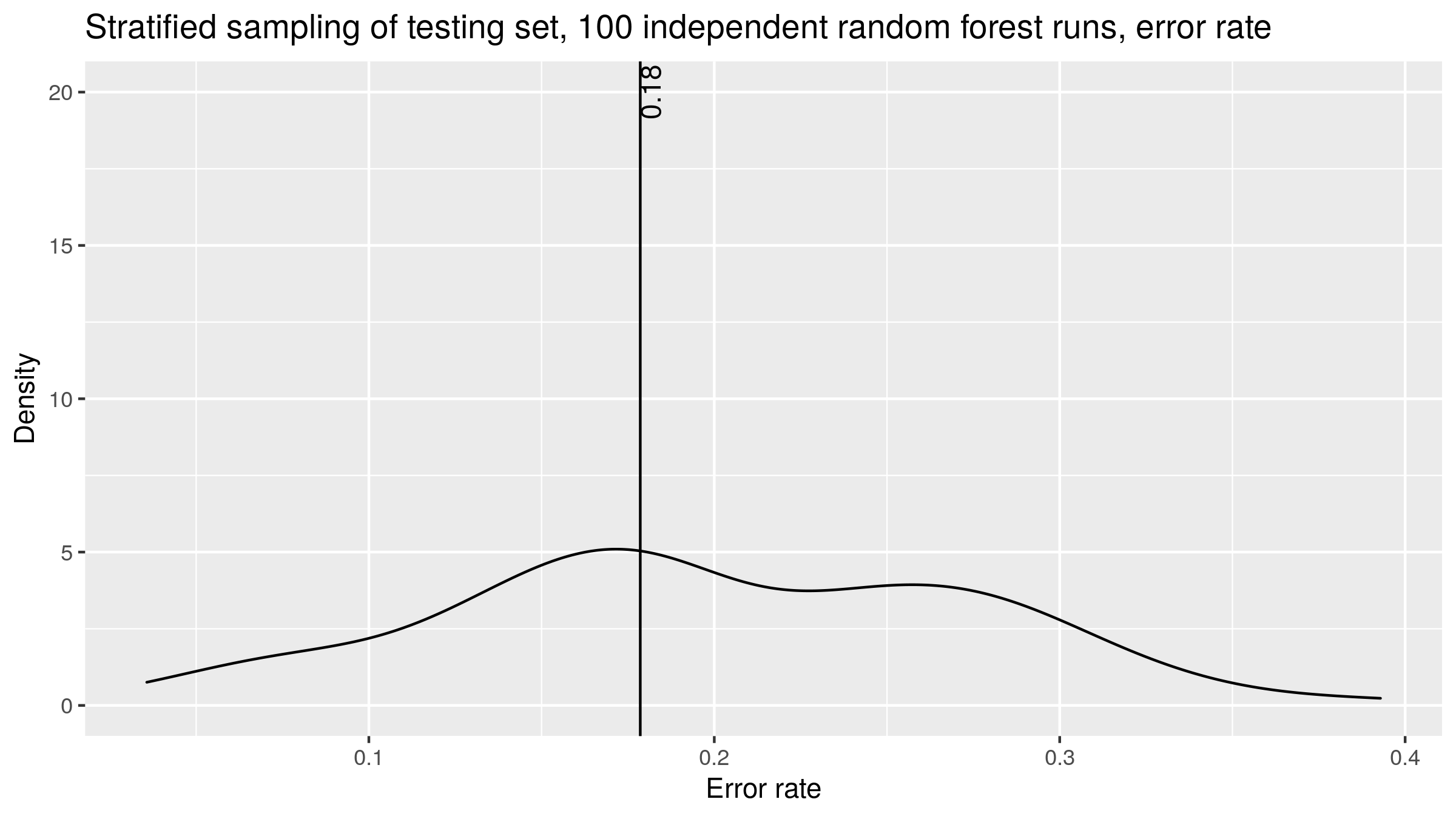


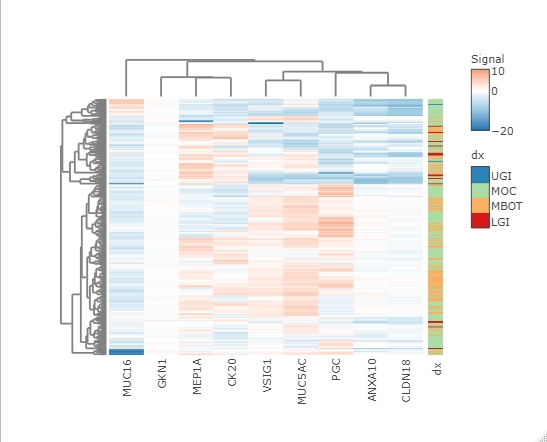
Figure S7: Heatmap of expression of the 9 selected classifier genes by tumor group (n=397)

Figure S8: Gene expression between tumour groups (n=363). Results of ANOVA with Tukey’s post-hoc test, p-values are adjusted for multiple comparisons. Box plot shows comparison of MEP1A expression between MOC and upper GI groups, broken down into gastric and pancreatic tumors.

| Gene | MOC-UGI | MOC-LGI |
| --- | --- | --- |
| ANXA10 |  |  |
| CLDN18 |  |  |
| ERBB2 |  |  |
| MEP1A |  |  |
| PD1 |  |  |
| DCN |  |  |
| TAGLN |  |  |
| THBS2 |  |  |
| MUC16 |  |  |
| SATB2 |  |  |
| PLA2R1 |  |  |
| PDL1 |  |  |
| MUC5AC |  |  |
| PGC |  |  |
| VSIG1 |  |  |
| GKN1 |  |  |
| CK20 |  |  |
| MUC2 |  |  |
| TYMS |  |  |


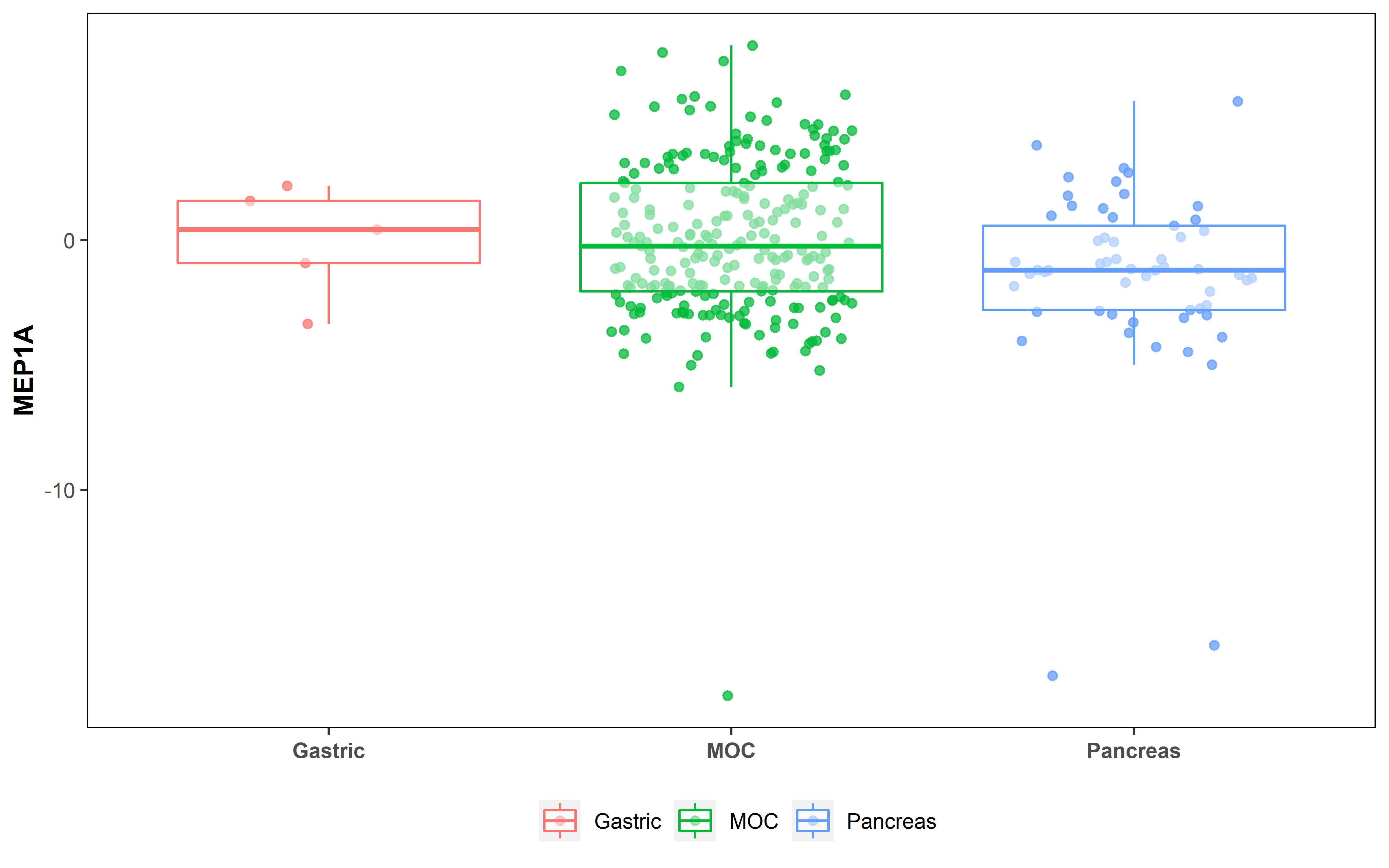


Figure S9: Box plots of gene expression of immune genes between tumor groups (n=360) PD-L1 (left) and PD-1 (right)


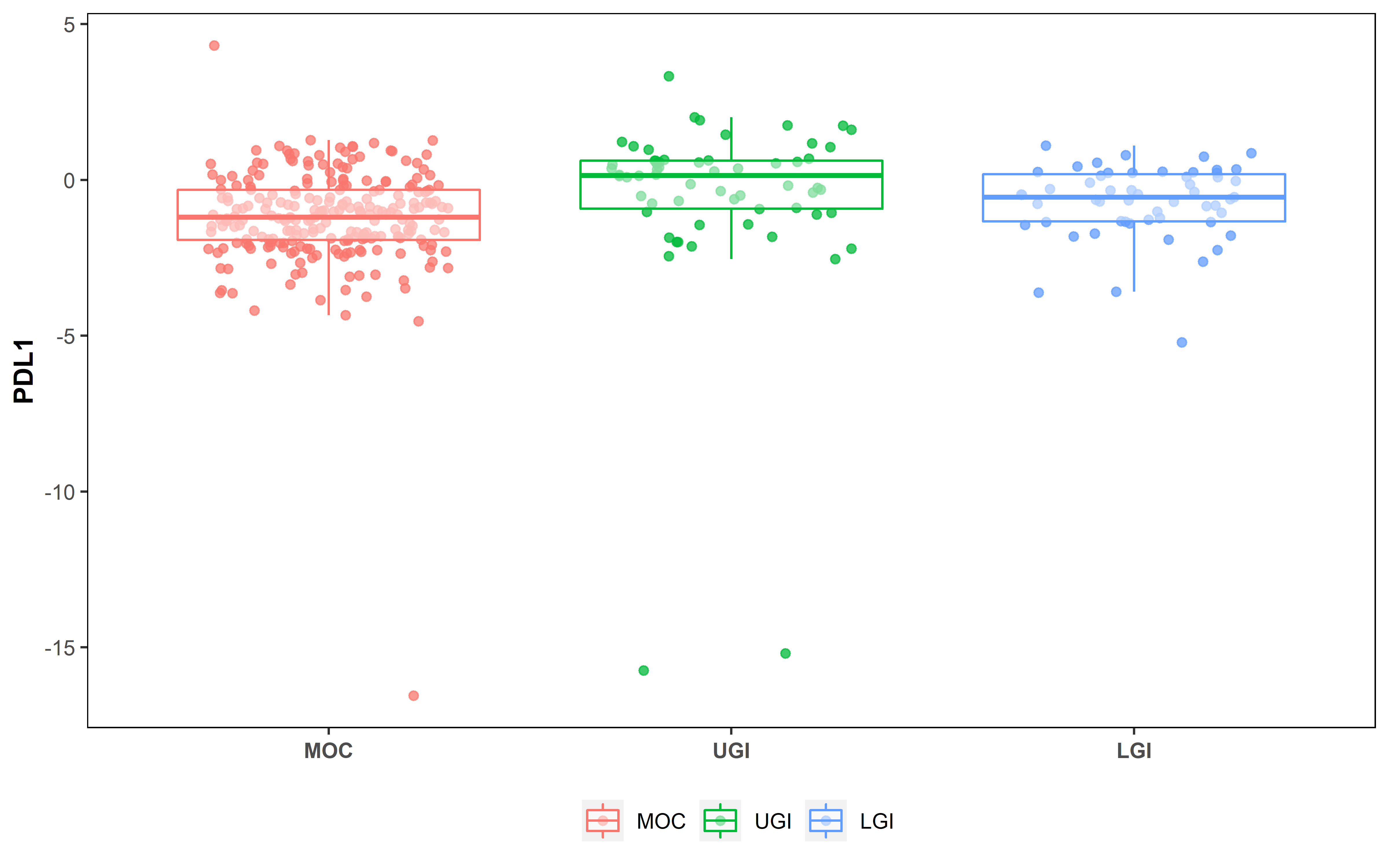


**p=0.03**


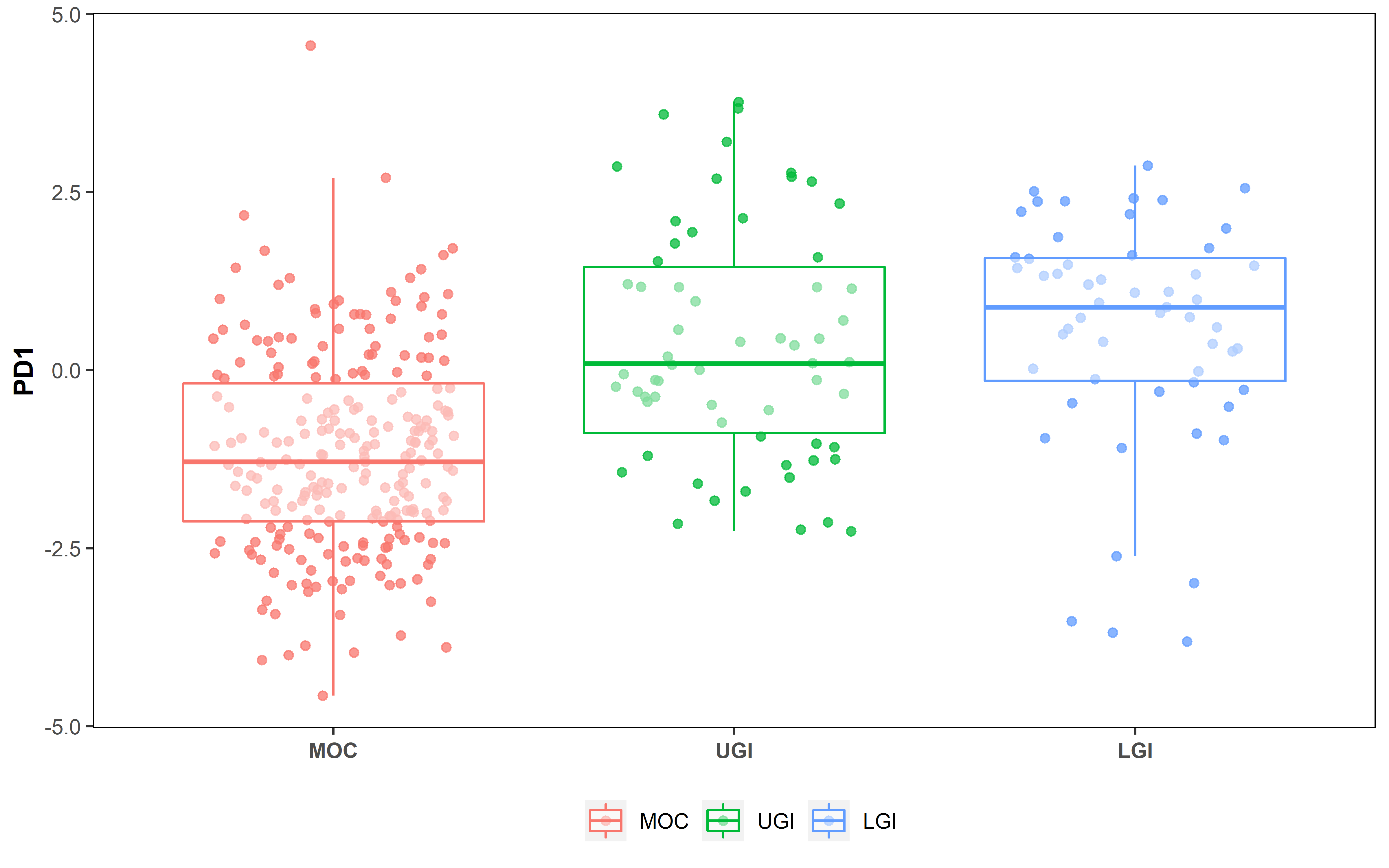


**p<0.0001**

**p<0.0001**

Figure S4: Spearman’s correlation between IHC and mRNA expression in A) HER2 (n=94), B) CK20 (n=178), C) SATB2 (n=308). Panel D shows HER2 mRNA expression by HER2 amplification (n=37).

Figure S10: (A) Violin plot of differential ERBB2 (HER2) expression between invasive tumor groups (n=360); (B) violin plot of differential HER2 expression between MBOT and stages of MOC (n=377)

1. B)


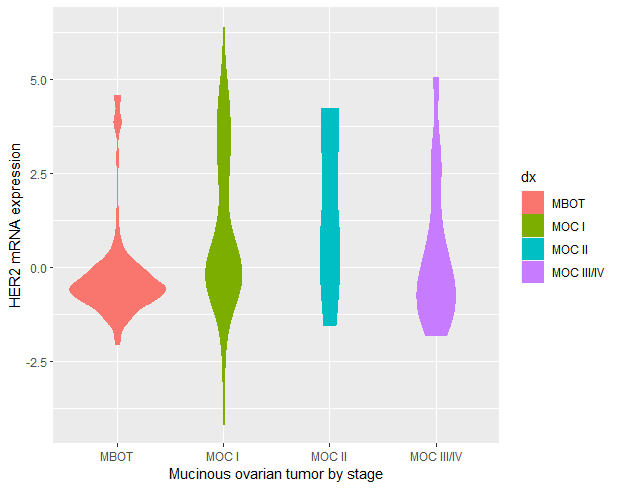


**p<0.001**

**p<0.0001**


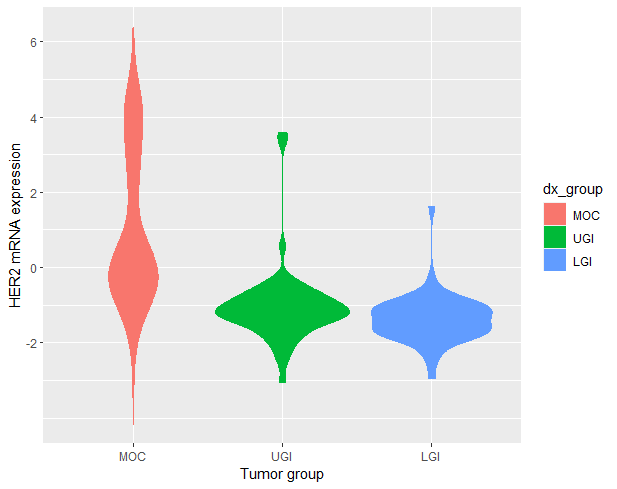


**p<0.0001**

**p<0.0001**

Figure S11: A: Box plot of HER2 mRNA expression by amplification status (n=37), B: correlation between mRNA expression and immunohistochemistry (IHC) scores (n=94)

1. B)


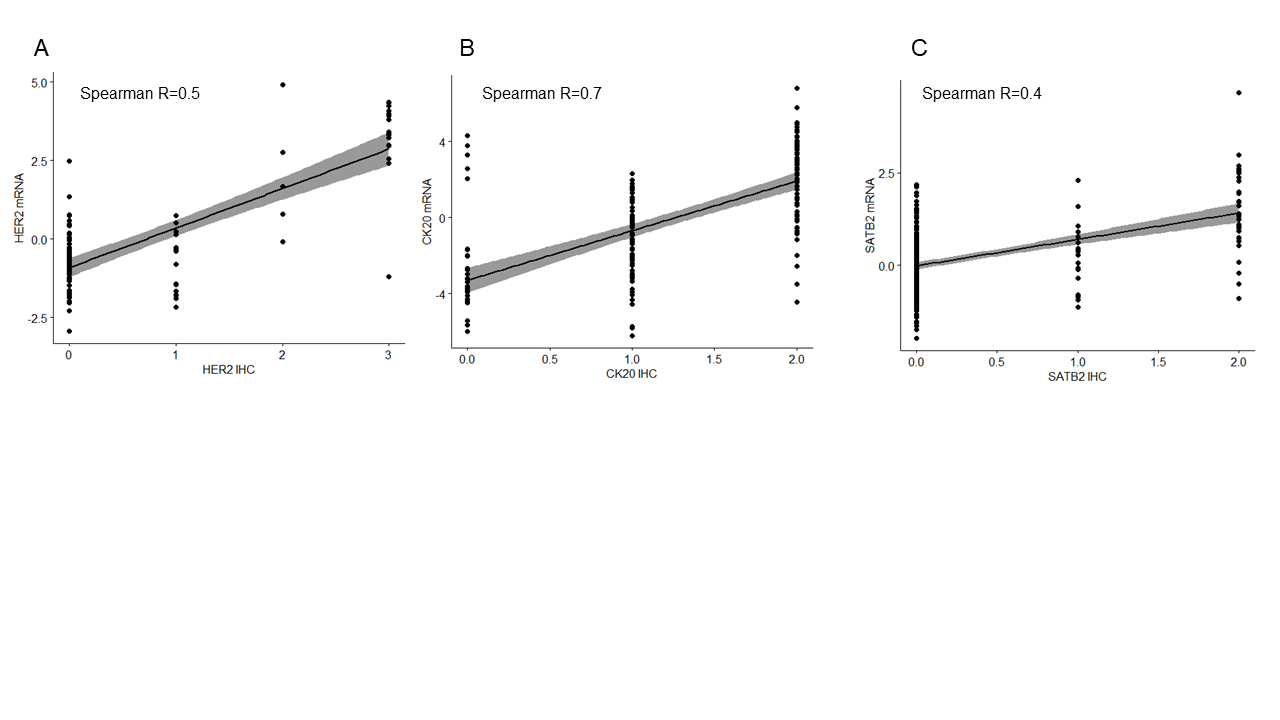

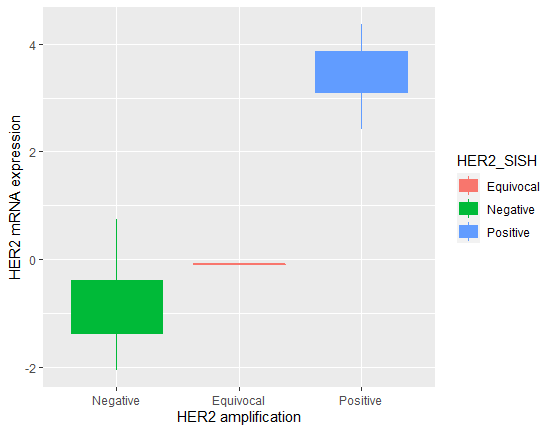


Figure S12: Kaplan Meier curve of overall survival of high mRNA expression/amplified versus low mRNA expression/non-amplified HER2 in MOC (n=233)


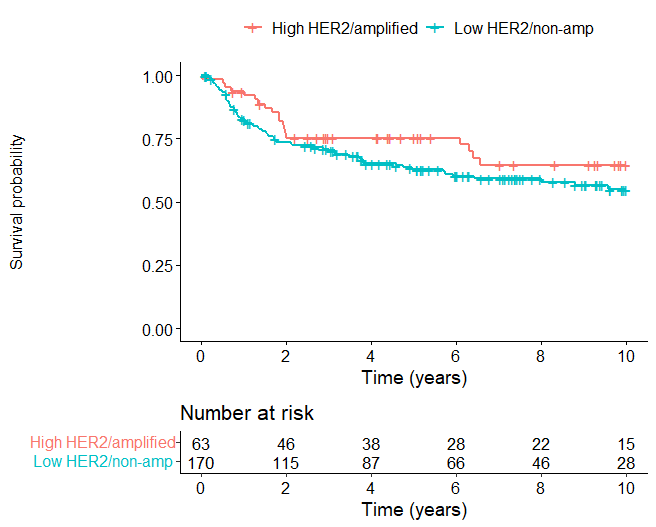


logrank p=0.2

Figure S13: Box plots of mRNA expression of TAGLN (left) and THBS2 (right) in MOC, grouped by increasing levels of tumor content in the samples.


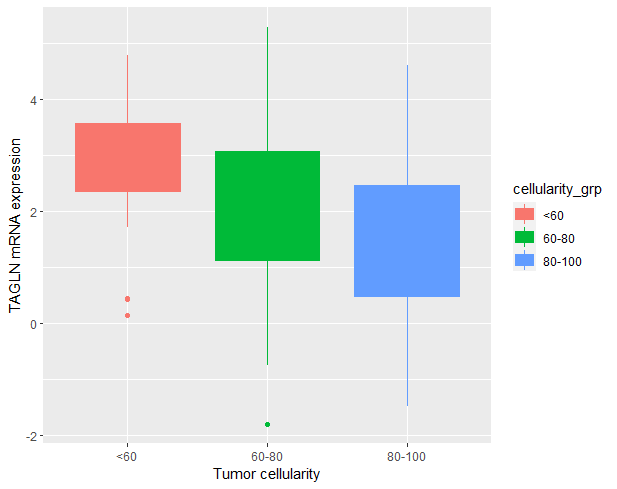

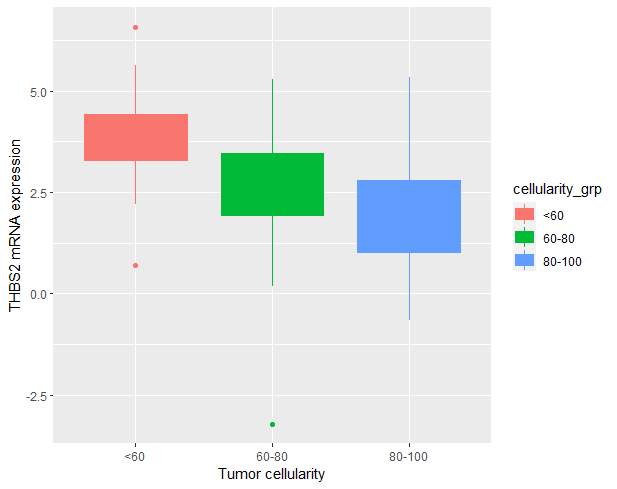

Supplement: Supplementary Figures S1-S13 [file ccr-22-1206_supplementary_figures_s1-s13_suppfs1-fs13.docx]
